# A preliminary study of a machine-learning prediction model for lung cancer diagnosis based on routine blood indicators and traditional chinese medicine diagnostic parameters

**DOI:** 10.3389/fphar.2026.1916858

**Published:** 2026-09-22

**Authors:** Zhanhui Lu, Chengcheng Zhang, Yiyi Liu, Jingru Qin, Lu Yang, Qianqian Yin, Zhijing Rao, Junqing Ji, Kaiting Chen, Wan Su, Xiangyang Zhan, Jingyun Jin, Wenbin Ben, Xingshuai Huang, Jincheng Liu, Guoying Wang, Zhiyi Zhou, Zhongqi Wang

**Affiliations:** 1 Department of Medical Oncology, Longhua Hospital, Shanghai University of Traditional Chinese Medicine, Shanghai, China; 2 Longhua Hospital, Cancer Institute, Shanghai University of Traditional Chinese Medicine, Shanghai, China; 3 Shanghai University of Traditional Chinese Medicine, Shanghai, China; 4 Integrated Chinese and Western Medicine, Shuguang Hospital affiliated to Shanghai University of Traditional Chinese Medicine, Shanghai, China; 5 School of Integrative Medicine, Shanghai University of Traditional Chinese Medicine, Shanghai, China; 6 China Pharmaceutical University, Nanjing, China; 7 Department of Anesthesiology, Shanghai ChangZheng hospital, Second Affiliated Hospital of Naval Medical University, Shanghai, China; 8 National Institute of Metrology, Center for Medical Metrology, Beijing, China; 9 Department of Critical Care Medicine, The Second People’s Hospital of Dongying, Dongying, Shandong, China

**Keywords:** lung cancer, machine learning, prediction model, routine blood indicators, tongue diagnosis, traditional chinese medicine

## Abstract

**Background:**

Early identification of lung cancer remains challenging in clinical practice, especially in patients who cannot undergo advanced imaging or invasive examinations. Traditional Chinese Medicine (TCM) provides distinctive diagnostic insights, but the potential role of its objective features in tumor screening remains insufficiently investigated. The objective of this study was to establish a multimodal machine-learning model combining routine laboratory examinations with selected objectified TCM indicators to screen for lung cancer.

**Methods:**

This real-world, retrospective cohort study included patients who visited the Departments of Oncology and General Surgery at Longhua Hospital, Shanghai University of Traditional Chinese Medicine, between January 2022 and December 2023. Following inclusion and exclusion screening, a total of 309 patients with pathologically confirmed lung cancer and 205 non-cancer patients were enrolled. All participants were randomly assigned using computer-generated randomisation in a 70:30 ratio to a training cohort (n = 358) and a validation cohort (n = 156). Five machine-learning algorithms and a logistic regression (LR) model were trained in the training cohort. Model performance in the validation cohort was assessed using area under the receiver operating characteristic curve (AUC), sensitivity, and specificity. SHAP analysis was applied to interpret feature contributions.

**Results:**

LASSO identified 13 key variables, encompassing age, blood biochemical indicators, objective tongue features, and TCM constitution scores. The XGBoost model constructed based on these variables demonstrated good discriminative performance for lung cancer in the validation set (AUC = 0.8745, 95% CI: 0.8219–0.9271; F1 = 0.7711). SHAP analysis revealed that age, absolute lymphocyte count, Qi-Deficiency Constitution Score (QDC-Score), apolipoprotein C3 (ApoC3), and tongue color features, including tongue coating color L* (TC-L) and full tongue color b* (FT-B), were the key factors driving model predictions.

**Conclusion:**

We developed a low-cost screening model that integrates TCM-related features with routine clinical parameters, demonstrating good accuracy for identifying high-risk individuals for lung cancer among non-tumor populations. This multimodal approach highlights the complementary value of integrating TCM indices with biomedical variables. Prospective multicenter validation is required to further optimize this model and facilitate its implementation in real-world clinical practice.

## Introduction

Lung cancer is the leading cause of cancer-related mortality worldwide (Siegel et al., 2025). With the development of targeted therapy, immunotherapy, and biological treatments, lung cancer mortality has shown a continuous decline in recent years (Aredo and Wakelee, 2024). However, lung cancer still accounts for the highest number of deaths, exceeding the combined mortality of colorectal, breast, and prostate cancers in 2022 (Siegel et al., 2025). Owing to its insidious onset, lung cancer is often diagnosed at an advanced stage when symptoms appear, with a 5-year relative survival rate of 27%. Early detection and timely standardized treatment can significantly improve patient survival (Siegel et al., 2025; Potter et al., 2022). Current routine lung cancer screening includes tumor biomarkers and chest CT, which greatly facilitate early detection, but their convenience and cost limit regular screening among the general population, especially in low- and middle-income countries (Crosby et al., 2022). Furthermore, individuals presenting with non-cancer illnesses—including fever, cholecystitis, or chronic gastritis—are seldom subjected to additional tumor marker testing in routine clinical visits. Therefore, establishing a simple, accessible, and economical method to screen high-risk individuals for lung cancer is necessary.

Traditional Chinese Medicine (TCM), as an adjunctive cancer therapy, improves quality of life and treatment outcomes through holistic approaches, offering advantages in reducing side effects and enhancing immunity at various stages of lung cancer treatment (Cao et al., 2017; Duan et al., 2018; Ning et al., 2018). TCM diagnosis is an indispensable component of TCM practice, consisting of inspection, listening/smelling, inquiry, and palpation. Among these methods, tongue diagnosis, TCM constitution classification, and pulse diagnosis have achieved higher levels of objectification. Previous studies have shown that tongue diagnosis is more accurate than biomarker testing for gastric cancer (Yuan et al., 2023), and prediction models based on tongue features can effectively distinguish healthy individuals, pulmonary nodules, and lung cancer patients (Shi et al., 2023a). TCM theory proposes that constitution is associated with an individual’s health status, disease susceptibility, and therapeutic outcomes (Su et al., 2008). Studies on constitution bias reported prevalences in breast cancer of 55.3% Yang-deficiency, 61.1% Yin-deficiency, 47.6% Blood-stasis, with 42.8% showing coexistence of all three constitutions (Lin et al., 2017). Constitution-type questionnaires derived from TCM theory may aid in disease diagnosis by assessing individual constitution. In TCM, pulse examination provides insight into health status, and differences in radial pulse waveforms have been observed between atrial fibrillation and non-atrial fibrillation patients (Cao et al., 2025). Studies further demonstrated that radial pulse waveforms can accurately detect early polycystic ovary syndrome (PCOS), but relevant investigations in the field of cancer are still lacking (Lim et al.).

Machine learning (ML) can identify cancer-related features within high-dimensional datasets using powerful statistical methods and construct classification models accordingly (Crosby et al., 2022), and previous studies have demonstrated that advanced ML algorithms such as Random Forest (RF), XGBoost, and LightGBM exhibit substantial potential in early disease prediction, as they can handle complex nonlinear relationships and high-dimensional data, thereby supporting early diagnosis and clinical intervention (Iwendi et al., 2020; Ogunleye and Wang, 2020; Ke et al., 2017). In oncology, ML models based on tongue image data outperform corresponding biomarker-based models (Yuan et al., 2023), and Shi constructed predictive ML models using tongue images and baseline data that were useful for estimating lung cancer risk, with subsequent studies further predicting disease staging using tongue features combined with blood biomarkers (Shi et al., 2023b).

Here, we applied ML for the first time to build a lung cancer diagnostic model incorporating tongue image features, constitution scores, pulse diagnosis, and routine blood tests, and evaluated its diagnostic performance, providing a novel approach for the scientific and standardized development of TCM diagnostic methods and offering more efficient and accurate tools for future clinical diagnosis.

This study was conducted on a retrospective cohort including 309 cancer patients and 205 non-cancer participants, from whom tongue images, TCM constitution questionnaires, pulse examination data, and routine blood indicators were collected, aiming to develop and validate an interpretable ML model for lung cancer prediction. The study implemented a logistic regression (LR) model and five ML algorithms, including Gradient Boosting Machine (GBM), XGBoost, Multi-Layer Perceptron (MLP), Random Forest (RF), and Support Vector Machine (SVM).

## Methods

### Design and patients

This study was a real-world retrospective cohort including lung cancer patients and non-cancer patients who visited the Oncology and General Surgery Departments of Longhua Hospital, Shanghai University of Traditional Chinese Medicine, from January 2022 to December 2023. After applying inclusion and exclusion criteria, 309 pathologically confirmed lung cancer patients and 205 non-cancer patients were included. These patients were randomly assigned at a 70:30 ratio into a training cohort (n = 358) and a validation cohort (n = 156). The training cohort was used to train and optimize models based on logistic regression and five machine learning (ML) algorithms, while the validation cohort was used to evaluate their predictive performance. The study protocol was approved by the Ethics Committee of Longhua Hospital, Shanghai University of Traditional Chinese Medicine, and complied with the Declaration of Helsinki (1975, revised 1983).

### Inclusion and exclusion criteria

All patients with lung cancer were diagnosed based on histopathological examination of biopsy or postoperative specimens, and histological classification was performed according to the fifth edition of the World Health Organization (WHO) Classification of Tumors of the Lung (2021). The control group comprised patients with benign lesions who visited the Department of General Surgery at Longhua Hospital, Shanghai University of Traditional Chinese Medicine, between January 2022 and December 2023. Malignancy was excluded based on a detailed medical history, chest computed tomography (CT) performed within 1 year, and postoperative pathological examination when clinically indicated. All participants underwent tongue image acquisition, radial pulse data collection from both wrists, and completion of the TCM constitution classification questionnaire.

The exclusion criteria were as follows: (1) concomitant malignancies of other types or a previous history of malignancy; (2) severe organic diseases or psychiatric disorders; and (3) food intake, tooth brushing, smoking, or other conditions that might affect tongue observation within 2 h before tongue image acquisition. The specific diagnostic composition of the control group is presented in Supplementary Table 1.

### Data and variables

Data collected included (1) demographic variables such as age and sex, (2) routine blood tests including complete blood count, liver and kidney function, D-dimer, and electrolytes, (3) tongue diagnosis images, (4) pulse diagnosis images, and (5) TCM constitution questionnaires. All data were collected and stored by trained researchers. To ensure accuracy, independent researchers conducted logical checks and re-evaluated the data. Smoking history is a well-established risk factor for lung cancer. However, as this was a retrospective study, smoking data were extracted from outpatient medical records, and completeness was limited. Among the 205 non-cancer controls, 79 (38.5%) had missing or unrecorded smoking status. Given this high proportion of missingness, inclusion of this variable would have compromised model stability and introduced information bias; therefore, it was excluded from the final predictive model.

### Blood tests collection

Blood test data were provided by the Central Laboratory of the Department of Laboratory Medicine at Longhua Hospital, Shanghai University of Traditional Chinese Medicine. Missingness in some laboratory indicators was mainly attributable to adjustments in the outpatient laboratory testing protocols. During the inclusion period from January 2022 to December 2023, the hospital optimized its routine outpatient laboratory test panels twice. Consequently, some tests, including triglycerides and cholesterol, were not routinely included in the standard laboratory panels and were performed only when specifically ordered by clinicians based on individual patient conditions. Therefore, the missingness of these indicators was not considered completely random and was characterized as missing not at random (MNAR) or missing at random (MAR).

Following the recommendations of Little (2014); Sterne et al. (2009), variables with more than 25% missing data were excluded because, even with multiple imputation, a high proportion of missing data may result in increased estimation bias and unstable imputation due to insufficient available information. Accordingly, variables with a missingness rate >25% were excluded to minimize potential information bias and ensure the reliability of subsequent imputation. For the variables retained in the analysis, the missingness rate was <25%, and missing values were imputed separately in the training and validation sets using a random forest-based imputation method.

To prevent data leakage, the complete dataset was first randomly divided into training and validation sets at a ratio of 70:30. Missing-value imputation was then performed independently within the two subsets. For both continuous and categorical variables, missing values were predicted and imputed using random forest models trained exclusively on the data within the corresponding subset. This strategy ensured that information from the validation set was not introduced into the imputation process of the training set, thereby improving the reliability of subsequent model evaluation. A total of 92 routine blood indicators were finally included, as listed in Supplementary Table 2.

### Tongue image data collection

Tongue images were acquired using a tongue diagnosis device (model: GMSX001) manufactured by Shanghai Guomin Health Company, equipped with a Sony IMX179 image sensor, enclosed light source, color temperature of 5,600 K, illumination of 1,200 lx, and color rendering index > 85 Ra. All acquired images were processed into JPG format. Each tongue was imaged at least twice. Images with fogging, underexposure, overexposure, coating staining, or abnormal tongue morphology were excluded. At the outpatient imaging site, trained research personnel performed the tongue image acquisition. The procedure was standardized: parameters were set, the instrument was disinfected with 75% medical alcohol, subjects placed their chin and forehead on support points, opened the mouth, extended and relaxed the tongue to flatten it; images were inspected to ensure full tongue surface visibility without fogging, light leakage, or exposure problems. Any inadequate image was retaken. Tongue color and texture features were extracted using the Nahefa Cloud System V2.0 developed by Shanghai Guomin Health Company. Following color calibration and image segmentation, the system automatically separated tongue body and coating regions. The tongue image quantification system was built using deep learning-based object detection (Wang et al., 2022), image segmentation (Chen et al., 2024; Chen et al., 2022; Chen et al., 2018), and image classification algorithms (Zhang et al., 2022; He et al., 2015; He et al., 2016). Each tongue image underwent repeated feature extraction, consistently generating 26 quantitative features, including tongue color, coating color, coating thickness, greasiness, petechiae, tooth-marks, fissures, and tongue size. Details are provided in Supplementary Table 3.

### Tongue region segmentation adopted DeepLabv3+ network with ResNet50 backbone

Tongue color classification module adopted ResNet18 lightweight convolutional neural network. The model training dataset contained 12,000 standardized clinical tongue images annotated by senior TCM physicians, with Adam optimizer (learning rate = 1e-4), batch size = 16, training epoch = 100, Dice loss function for segmentation tasks, cross-entropy loss for color classification tasks. All feature extraction logic was solidified inside the Nahefa Cloud System V2.0, and model weight files were fixed without iterative updates during subject data collection.

Unified strict pre-collection subject control protocols were implemented for all participants: 1) All subjects fasted for at least 2 h before tongue image acquisition; 2) No smoking, coffee, colored drinks, pigmented food within 2 h before shooting; 3) Subjects stopped taking oral pigmented traditional Chinese patent medicines 12 h before data collection; 4) Tooth brushing, mouthwash and tongue scraping were prohibited within 1 h before image capture to avoid altering tongue coating and tongue body color.

### Pulse diagnosis data collection

The pulse diagnosis was performed using a device manufactured by Shanghai Guomin Health Company, whose pulse waveform algorithm is based on Professor Fei Zhaofu’s theory of modern TCM pulse diagnosis (Fei, 2012). This algorithm is a classical and widely used pulse diagnosis approach. The pulse waveform is shown in Figure 1. Temporal and amplitude features were extracted from the waveform, followed by feature-based classification to generate the final pulse output. The device captured the pulse features objectively and comprehensively, producing 8 diagnostic elements such as pulse position and pulse strength. Details are provided in Supplementary Table 4.

**FIGURE 1:**
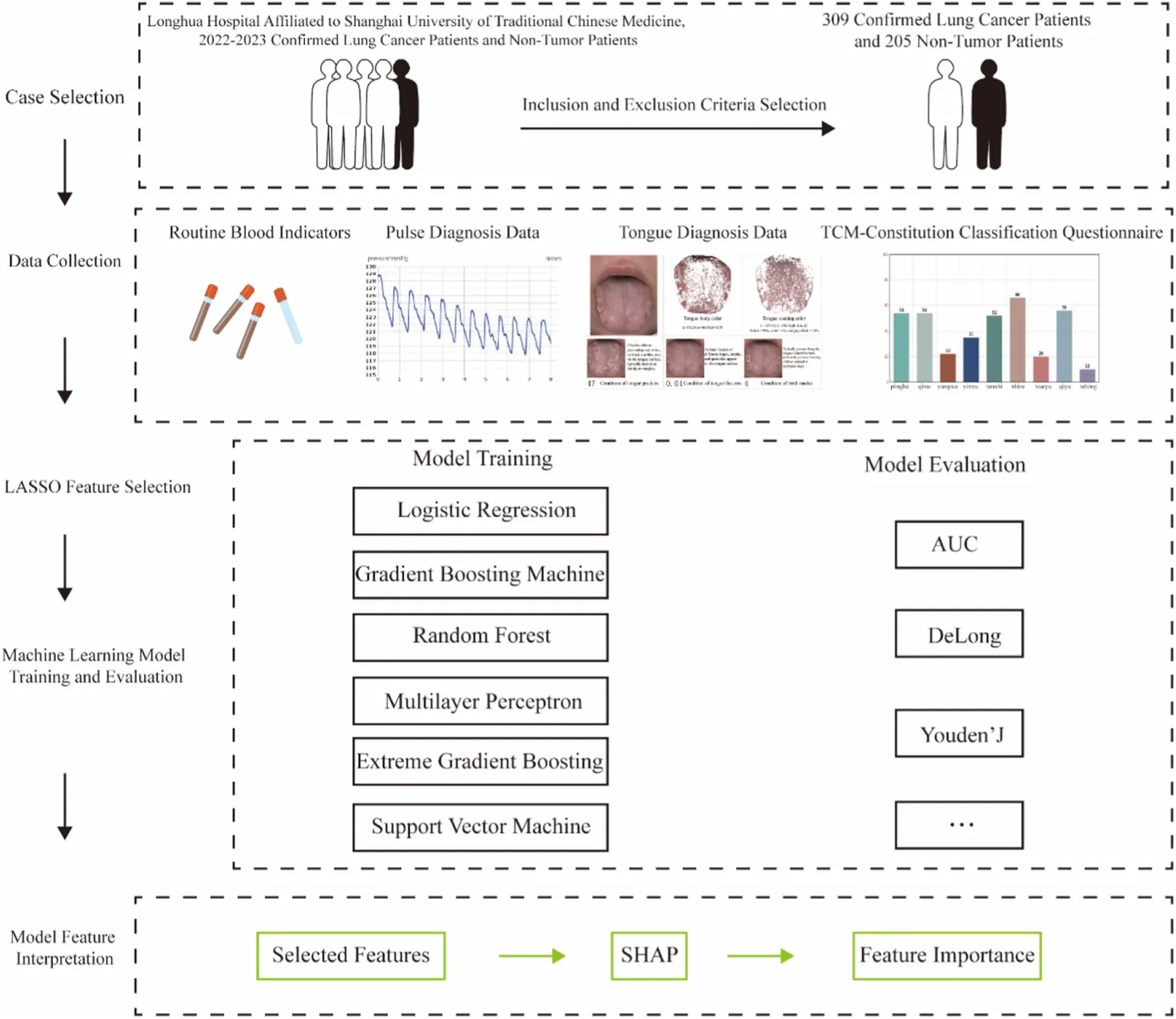
Flow chart of this study.

### TCM constitution scoring

The constitution scores were determined according to the “TCM Constitution Classification and Assessment Questionnaire” (Supplementary Material 2) (Wang and Jin, 2012). Each item was rated on a five-point scale, and both raw and transformed scores were calculated. The transformed scores represented nine constitution types: Pinghe, Qi-deficiency, Yang-deficiency, Yin-deficiency, Phlegm-dampness, Damp-heat (F-type), Blood stasis, Qi-stagnation, and Special constitution. Explanations of the constitution types are provided in Supplementary Table 5. Since less than 75% of the items for the special constitution were completed, this score was excluded.

Raw score=sum of item scores

$$\text{Converted score}=\left[\left(\text{raw score}-\text{number of items}\right)\;/\;\left(\text{number of items}\times 4\right)\right]\times 100$$

### Modeling and validation

To reduce dimensionality and identify the most informative predictors, we applied Least Absolute Shrinkage and Selection Operator (LASSO) logistic regression with 10-fold cross-validation exclusively within the training cohort. The regularization parameter λ was selected using the one-standard-error rule (λ.1se), which retains the most parsimonious model within one standard error of the minimum cross-validation error, thereby controlling feature selection, reducing overfitting, and improving generalizability. Variables with non-zero coefficients were retained as the final candidate features.Six machine-learning classifiers—Logistic Regression (LR), Gradient Boosting Machine (GBM), Random Forest (RF), Multilayer Perceptron (MLP), Extreme Gradient Boosting (XGBoost), and Support Vector Machine (SVM)—were subsequently trained and hyperparameter-tuned. To ensure robust internal validation, repeated stratified 10-fold cross-validation (10 repetitions) was performed exclusively within the training cohort for model optimization. In addition, bootstrap resampling with 1,000 iterations was performed to obtain bias-corrected 95% confidence intervals (CIs) for the area under the receiver operating characteristic curve (AUC).Model discrimination was evaluated using the AUC, while diagnostic performance was assessed using sensitivity, specificity, positive predictive value (PPV), negative predictive value (NPV), accuracy, and F1 score. The Youden index was used to determine the optimal cutoff value that balanced sensitivity and specificity. Differences in AUCs between models were compared using the DeLong test.

### Model interpretation using SHAP

To elucidate the contribution of individual predictors to model behaviour, we employed the SHapley Additive exPlanations (SHAP) framework, which provides a unified, axiomatic measure of feature attribution grounded in cooperative game theory. SHAP decomposes each model prediction into additive contributions from all input variables, thereby enabling both local interrogation of individual predictions and global assessment of feature influence. SHAP values were estimated using the fastshap and shapviz packages in R, which approximate Shapley values through model-agnostic perturbation of predictor space. Global feature relevance was quantified as the mean absolute SHAP value across all observations.

## Statistics and implementation

The clinical dataset was processed and analyzed using R version 4.2.1. For normally distributed variables, data were expressed as mean ± standard deviation (SD), whereas non-normally distributed variables were reported as median with interquartile range. Student’s t-test and chi-square test were used to evaluate differences in continuous and categorical variables between the two groups, respectively. A p-value < 0.05 was considered statistically significant.

## Results

### Patient’s basic characteristics

The median age of the entire cohort was 66.00 [57.00, 71.00] years (Table 1). The sex distribution was comparable between the two groups (p > 0.05), whereas a significant age difference was observed (p < 0.01), with non-lung cancer patients being considerably younger than lung cancer patients. Among the 205 non-cancer controls, the majority presented with benign hepatobiliary, gastrointestinal, or metabolic comorbidities that prompted their hospital visits (Supplementary Table 1). Hepatobiliary diseases were the most prevalent, affecting 114 participants (55.6%), with cholecystitis (35.6%) and cholelithiasis (9.8%) being the leading diagnoses. Gastrointestinal conditions were observed in 44 participants (21.5%), predominantly chronic gastritis (9.3%) and appendicitis (4.4%). Endocrine or metabolic-related benign findings, including thyroid nodules, lipomas, and type 2 diabetes mellitus, were documented in 27 participants (13.2%). Additional diagnoses comprised hernia (5.4%), renal diseases (3.4%), and cardiovascular disease (0.5%). Notably, all these conditions were non-malignant and clinically distinct from lung cancer, providing a clinically relevant benign comparison group. Given the real-world nature of this study population and the established predictive role of age in cancer, we retained the observed age distribution rather than performing *post hoc* propensity score matching (PSM). Other clinical indicators are provided in Supplementary Tables 1,6,7.

**TABLE 1:** Baseline characteristics of the study participants.

Variables	Total (n = 514)	lung_cancer (n = 309)	non_cancer (n = 205)	*P*
Age, median (Q1,Q3)	66 (57, 71)	67 (61, 71)	60 (47, 69)	< 0.001
Sex, n (%)	​	​	​	0.918
Female	281 (55)	170 (55)	111 (54)	​
Male	233 (45)	139 (45)	94 (46)	​

Smoking history was unavailable for 38.5% of the total enrolled participants owing to incomplete medical records or patient-reported data.

### Development of models

Using LASSO logistic regression with 10-fold cross-validation, we identified a parsimonious subset of predictive features from the full pool of candidate variables. To enhance model sparsity and reduce overfitting, a more conservative penalty (2×λ_1se_) was applied. Under this strict constraint, 13 predictors retained non-zero coefficients, indicating stable contributions to the outcome (Figures 2A,B). These features included age; Tongue Coating Color – L (TC-L), representing the lightness of the tongue coating in the CIE-Lab color space, with lower values indicating darker tones and higher values indicating lighter tones; Degree of White Coating (TC-White), representing the degree of white coating on the tongue surface; Tongue Body Color – L (TB-L), representing the lightness of the tongue body in the CIE-Lab color space; Full Tongue Color – b* (FT-B), representing the blue–yellow axis of the entire tongue in the CIE-Lab color space, with higher values indicating more yellowish tones and lower values indicating more bluish tones; Qualitative Tongue Color Classification (TCC), a categorical classification of tongue-body color, with TCC4 denoting the purple tongue-body color category (code 4); Quantitative Coating Thickness Index (CT-Quant), representing the quantitative index of tongue-coating thickness; Qi-Deficiency Constitution Score (QDC-Score), a quantitative score reflecting qi-deficiency constitution; Blood-Stasis Constitution Score (BSC-Score), a quantitative score reflecting blood-stasis constitution; Gamma-Glutamyl Transferase (GGT); Apolipoprotein C3 (ApoC3); Fibronectin (FN); and absolute lymphocyte count (LYM#).These 13 clinical features were used as predictors for model construction, and were subsequently incorporated into logistic regression and five machine learning models.

**FIGURE 2:**
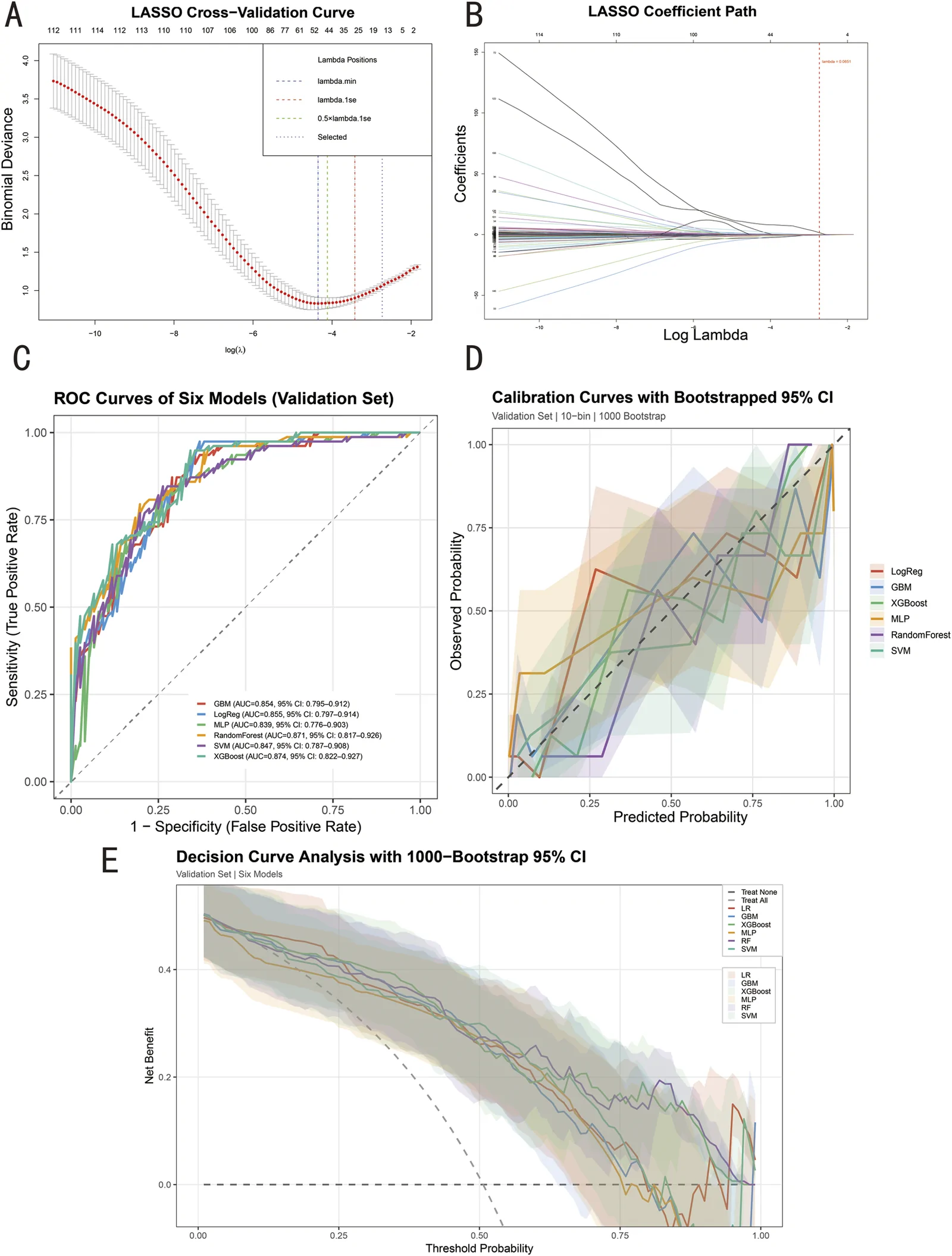
**(A)** The Least Absolute Shrinkage and Selection Operator (LASSO) coefficient profiles of all variables; **(B)** Selection of the optimal tuning parameter (lambda) in the LASSO model; **(C)** Receiver operating characteristic (ROC) curves of the six machine learning (ML) models for predicting lung cancer; **(D)** Calibration curves of the predictive models; **(E)** Clinical decision curve analysis (DCA) for each model.

### Validation of models

This study employed Logistic Regression (LR), Gradient Boosting Machine (GBM), Random Forest (RF), Multilayer Perceptron (MLP), Extreme Gradient Boosting (XGBoost), and Support Vector Machine (SVM) for lung cancer prediction. The ROC curves and calibration curves for each model are presented in Figure 2. Evaluation metrics included accuracy, F1 score, sensitivity, specificity, positive predictive value (PPV), negative predictive value (NPV), AUC, and PR-AUC.

As shown in Table 2, XGBoost achieved the highest AUC among the evaluated models, with an AUC of 0.8745 (95% confidence interval [CI]: 0.8219–0.9271), followed closely by Random Forest, which achieved an AUC of 0.8715 (95% CI: 0.8171–0.9258). The confidence intervals of the two models overlapped, suggesting comparable overall discriminative performance, with both models demonstrating relatively high discrimination. In terms of the area under the precision–recall curve (PR AUC), Random Forest (0.8813) and XGBoost (0.8803) also ranked first and second, respectively. Regarding overall classification performance, Support Vector Machine achieved the highest F1 score (0.8049), as well as the highest accuracy and Kappa coefficient (0.7922 and 0.5838, respectively), indicating robust overall classification performance. Random Forest achieved an F1 score of 0.7882 and the highest sensitivity among all models (0.8590), but had relatively lower specificity (0.6711), suggesting a greater tendency toward identifying positive cases and potentially offering advantages in specific clinical scenarios where minimizing missed diagnoses is prioritized. Gradient Boosting Machine achieved an F1 score of 0.7879 and an AUC of 0.8537 (95% CI: 0.7953–0.9122), demonstrating stable classification performance.XGBoost achieved the highest Youden index (0.6066), indicating a favorable balance between sensitivity and specificity, which were 0.8205 and 0.6842, respectively. Logistic Regression and Support Vector Machine also demonstrated reliable classification performance, with AUCs of 0.8551 (95% CI: 0.7966–0.9136) and 0.8473 (95% CI: 0.7867–0.9079), respectively. Notably, Logistic Regression achieved the highest specificity (0.7237), suggesting a potential advantage in reducing false-positive classifications. Multi-layer Perceptron showed relatively weaker performance, with an AUC of 0.8392 (95% CI: 0.7758–0.9026) and an F1 score of 0.7805, with its overall evaluation metrics being slightly lower than those of the other models.

**TABLE 2:** Model evaluation metrics.

Model	Accuracy	Kappa	Sensitivity	Specificity	PPV	NPV	F1	AUC	AUC_CI_lower	AUC_CI_upper	PR_AUC	Youden_Index	Optimal_Threshold
XGBoost	0.7532	0.5056	0.8205	0.6842	0.7273	0.7879	0.7711	0.8745	0.8219	0.9271	0.8803	0.6066	0.345
RandomForest	0.7662	0.5313	0.859	0.6711	0.7283	0.8226	0.7882	0.8715	0.8171	0.9258	0.8813	0.5975	0.603
LR	0.7532	0.5061	0.7821	0.7237	0.7439	0.7639	0.7625	0.8551	0.7966	0.9136	0.8547	0.6059	0.2072
GBM	0.7727	0.5447	0.8333	0.7105	0.7471	0.806	0.7879	0.8537	0.7953	0.9122	0.8539	0.5823	0.4483
SVM	0.7922	0.5838	0.8462	0.7368	0.7674	0.8235	0.8049	0.8473	0.7867	0.9079	0.853	0.583	0.5065
MLP	0.7662	0.5318	0.8205	0.7105	0.7442	0.7941	0.7805	0.8392	0.7758	0.9026	0.8137	0.5705	0.5744

LR, logistic regression; GBM, gradient boosting machine; XGBoost, eXtreme Gradient Boosting; MLP, multilayer perceptron; RF, random forest; SVM, support vector machine; PPV, positive predictive value; NPV, negative predictive value; AUC, area under the receiver operating characteristic curve; PR_AUC, Area Under the Precision-Recall Curve.

To obtain robust estimates of model discrimination, we performed 1,000 bootstrap resampling iterations. As shown in Table 3, XGBoost achieved the highest bias-corrected mean AUC of 0.8745 (95% CI: 0.8163–0.9247), followed by Random Forest, with a mean AUC of 0.8707 (95% CI: 0.8134–0.9221). The bootstrap standard deviations were similar between the two models (XGBoost: 0.0278; Random Forest: 0.0284), indicating relatively stable performance estimates. Notably, the 95% CIs of XGBoost and Random Forest overlapped substantially, and the DeLong test confirmed that the difference in AUC between the two models was not statistically significant (*P* = 0.832). Among the other classifiers, Logistic Regression and Gradient Boosting Machine (GBM) achieved mean AUCs of 0.8541 and 0.8529, respectively, indicating moderate discriminative performance. Multi-layer Perceptron (MLP) had the lowest mean AUC at 0.8388 (95% CI: 0.7736–0.9004).

**TABLE 3:** Bootstrap resampling results.

Model	Bootstrap Mean AUC	Bootstrap SD	95% CI	Original AUC
XGBoost	0.8745	0.0278	0.8163–0.9247	0.8745
Random forest	0.8707	0.0284	0.8134–0.9221	0.8715
Logistic regression	0.8541	0.0297	0.7955–0.9069	0.8551
GBM	0.8529	0.0302	0.7905–0.9084	0.8537
SVM	0.8465	0.0308	0.7835–0.9028	0.8473
MLP	0.8388	0.0326	0.7736–0.9004	0.8392

Among the six models evaluated, XGBoost achieved the highest bias-corrected mean AUC after bootstrap resampling (0.8745, 95% CI: 0.8163–0.9247) and had the highest Youden index (0.6066) among all models, indicating the most favorable balance between sensitivity and specificity. Random Forest achieved a bootstrap mean AUC of 0.8707 (95% CI: 0.8134–0.9221), with similar bootstrap standard deviations between the two models (0.0278 and 0.0284, respectively), indicating relatively stable performance estimates for both models. The DeLong test further showed no statistically significant difference in AUC between XGBoost and Random Forest (*P* = 0.832), suggesting comparable overall discriminative performance.

Random Forest showed slightly higher sensitivity (0.8590) and PR AUC (0.8813) than XGBoost (0.8205 and 0.8803, respectively), whereas Support Vector Machine achieved the highest F1 score (0.8049) and accuracy (0.7922). Notably, the bootstrap 95% CIs showed substantial overlap among the AUC estimates of the different models, and pairwise DeLong tests (Table 4) confirmed that none of the differences in AUC between models reached statistical significance (all *P* > 0.05), indicating that the overall discriminative performance of the models was broadly comparable.

**TABLE 4:** The AUC DeLong test results.

Model1	Model2	AUC1	AUC2	Difference	P_value	Significant
RandomForest	SVM	0.8715	0.8473	0.0241	5.925e-02	No
MLP	RandomForest	0.8392	0.8715	−0.0322	1.305e-01	No
GBM	XGBoost	0.8537	0.8745	−0.0207	1.359e-01	No
GBM	RandomForest	0.8537	0.8715	−0.0177	1.592e-01	No
XGBoost	MLP	0.8745	0.8392	0.0353	1.64e-01	No
XGBoost	SVM	0.8745	0.8473	0.0272	1.737e-01	No
LogReg	RandomForest	0.8551	0.8715	−0.0164	3.101e-01	No
LogReg	XGBoost	0.8551	0.8745	−0.0194	3.467e-01	No
LogReg	MLP	0.8551	0.8392	0.0159	3.885e-01	No
GBM	MLP	0.8537	0.8392	0.0145	5.261e-01	No
LogReg	SVM	0.8551	0.8473	0.0078	5.936e-01	No
MLP	SVM	0.8392	0.8473	−0.0081	6.56e-01	No
GBM	SVM	0.8537	0.8473	0.0064	7.096e-01	No
XGBoost	RandomForest	0.8745	0.8715	0.003	8.321e-01	No
LogReg	GBM	0.8551	0.8537	0.0013	9.37e-01	No

Model comparisons were performed using DeLong’s test to evaluate the statistical significance of differences in the area under the curve (AUC). Abbreviations: AUC, area under the curve; RF, random forest; LR, logistic regression; GBM, gradient boosting machine; XGBoost, Extreme Gradient Boosting; MLP, multilayer perceptron; SVM, support vector machine.

Considering the bootstrap-corrected AUC as the primary evaluation endpoint, the overall diagnostic performance reflected by the Youden index, and clinical utility, XGBoost was selected as the optimal predictive model in this study, while Random Forest was considered a performance-comparable alternative, particularly for screening scenarios in which a higher positive detection rate is prioritized. Calibration curves demonstrated good calibration across all models, particularly for XGBoost and Random Forest (Figure 2D). Quantitative calibration assessment further supported the satisfactory performance of the optimal XGBoost model. The Brier score was 0.146, corresponding to a Brier skill score of 0.417 relative to the no-information model, indicating good probabilistic accuracy. The calibration intercept was −0.132 and the calibration slope was 1.208 (ideal = 1), suggesting only minor deviation from perfect calibration. The Hosmer–Lemeshow goodness-of-fit test yielded χ^2^ = 13.88 (df = 8, *P* = 0.085), indicating no statistically significant evidence of lack of fit at the 0.05 level. Collectively, these findings indicate that the XGBoost model demonstrated favorable discrimination, classification performance, and calibration, supporting its potential utility as a low-cost predictive tool for lung cancer risk stratification.

Decision curve analysis demonstrated that all six models provided positive net benefits across clinically relevant threshold probabilities ranging from 10% to 30% (Figure 2E). Detailed net benefit estimates with corresponding 1,000-bootstrap 95% confidence intervals at threshold probabilities of 10%, 15%, 20%, 25%, and 30% are provided in Supplementary Table 8. At a threshold probability of 10%, Logistic Regression showed the highest net benefit (0.468, 95% CI: 0.386–0.551), closely followed by XGBoost (0.465, 95% CI: 0.380–0.549). At a 20% threshold probability, corresponding to a moderate-risk screening context, XGBoost achieved a net benefit of 0.430 (95% CI: 0.341–0.520), indicating a favorable clinical net benefit compared with the treat-all strategy. This was comparable to Logistic Regression (0.449, 95% CI: 0.362–0.537) and Random Forest (0.423, 95% CI: 0.330–0.515) at the same threshold. At a 30% threshold probability, XGBoost maintained the highest net benefit among all models (0.403, 95% CI: 0.307–0.497), exceeding that of Gradient Boosting Machine (0.386, 95% CI: 0.294–0.479) and Random Forest (0.388, 95% CI: 0.290–0.483). Across the entire 10%–30% threshold range, the lower bounds of the 1,000-bootstrap 95% CIs for all six models remained above the treat-none strategy, supporting their potential clinical utility across this range of decision thresholds.

Collectively, these findings indicate that the XGBoost model demonstrated favorable discrimination, classification performance, calibration, and clinical net benefit, supporting its potential utility as a low-cost predictive tool for lung cancer risk stratification.

To evaluate the contribution of different feature types to model predictive performance, we constructed five XGBoost models based on different feature combinations: the full-feature model (Model 1: clinical features + objective tongue features + blood indicators), the clinical plus tongue-feature model (Model 2), the tongue-feature plus blood-indicator model (Model 4), the clinical plus blood-indicator model (Model 3), and the clinical baseline model (Model 5: clinical features only). Figure 3 and Tables 5, 6 present the predictive performance of these models in the test set, together with the bias-corrected results obtained from 1,000 bootstrap resampling iterations.

**FIGURE 3:**
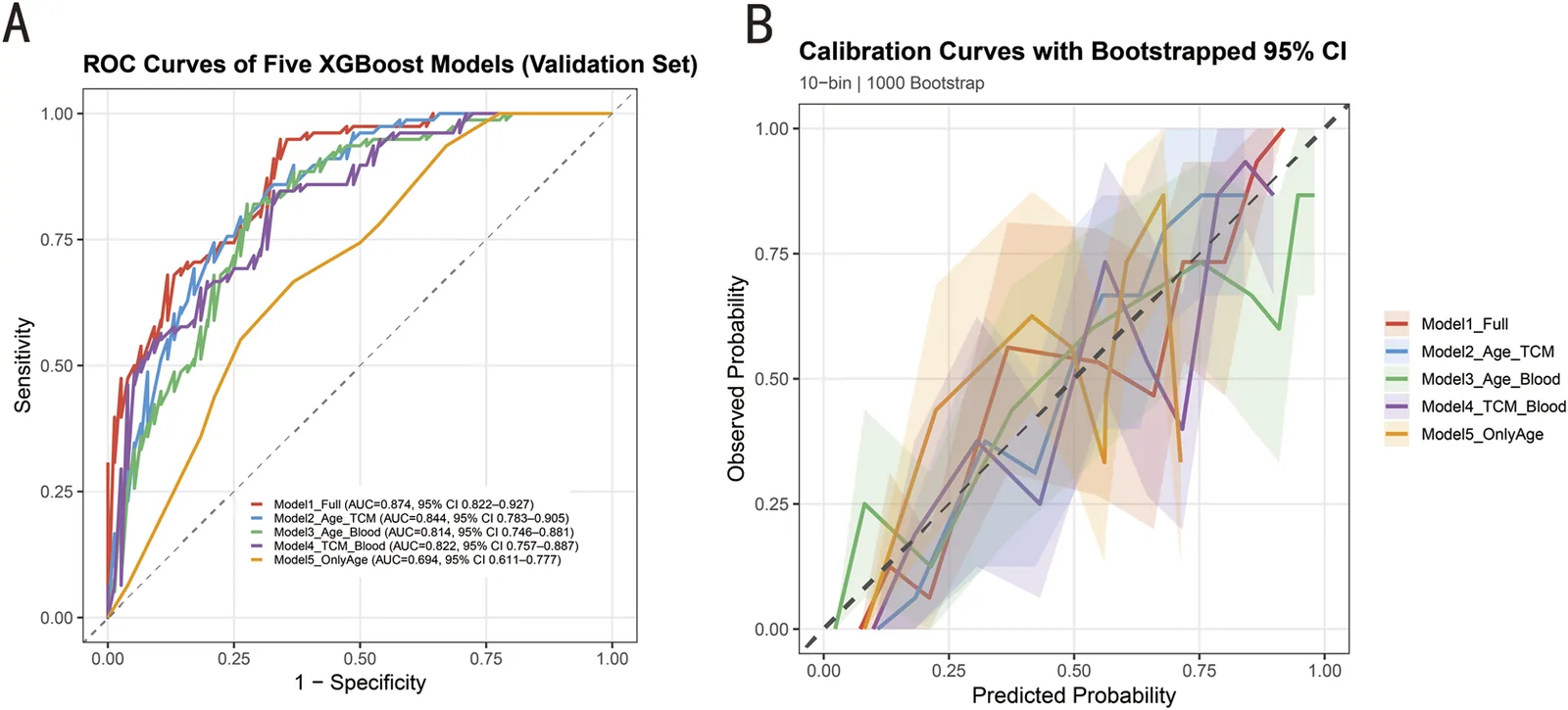
**(A)** Comparison of XGBoost models with different features; **(B)** Calibration curves of the predictive models with different features.

**TABLE 5:** Performance of XGBoost models with different feature combinations in the test set.

Model	N	Accuracy	Kappa	Sensitivity	Specificity	PPV	NPV	F1	AUC (95% CI)	PR-AUC
Full model (clinical + TCM + blood)	13	0.7532	0.5056	0.8205	0.6842	0.7273	0.7879	0.7711	0.8745 (0.8219–0.9271)	0.8803
Clinical + TCM	5	0.7662	0.5321	0.7949	0.7368	0.7561	0.7778	0.775	0.8439 (0.7827–0.9051)	0.8245
TCM + blood	12	0.7597	0.5183	0.8462	0.6711	0.7253	0.8095	0.7811	0.8220 (0.7571–0.8870)	0.81
Clinical + blood	9	0.7727	0.5448	0.8205	0.7237	0.7529	0.7971	0.7853	0.8138 (0.7461–0.8815)	0.7862
Clinical only (age)	1	0.6234	0.2443	0.7436	0.5	0.6042	0.6552	0.6667	0.6939 (0.6112–0.7766)	0.6496

Abbreviations: PPV, positive predictive value; NPV, negative predictive value; AUC, area under the receiver operating characteristic curve.

**TABLE 6:** Bootstrap validation (1000 iterations) of XGBoost models with different feature combinations.

Model	Bootstrap AUC (95% CI)	Bootstrap SD
Full model (clinical + TCM + blood)	0.8745 (0.8163–0.9247)	0.0278
Clinical + TCM	0.8445 (0.7757–0.9049)	0.0324
TCM + blood	0.8215 (0.7529–0.8810)	0.0335
Clinical + blood	0.8127 (0.7377–0.8766)	0.0348
Clinical only (age)	0.6926 (0.6070–0.7695)	0.0427

Overall, Model 1 (full-feature model) demonstrated the best performance. Its bootstrap bias-corrected mean AUC was 0.8745 (95% CI: 0.8163–0.9247), with a bootstrap standard deviation of 0.0278, representing the most stable performance estimate among all models. The PR AUC was 0.8803 and the Youden index was 0.6066, indicating strong discriminative performance in distinguishing patients with lung cancer from non-cancer individuals and a favorable balance between sensitivity and specificity. The classification metrics were also robust, with an accuracy of 0.7532, F1 score of 0.7711, sensitivity of 0.8205, and specificity of 0.6842.

In comparison, Model 2 (clinical + tongue features) achieved the second-highest bootstrap mean AUC of 0.8445 (95% CI: 0.7757–0.9049). The bootstrap mean AUCs of Model 4 (tongue features + blood indicators) and Model 3 (clinical + blood indicators) were 0.8215 (95% CI: 0.7529–0.8810) and 0.8127 (95% CI: 0.7377–0.8766), respectively, indicating a progressive decrease in performance. Model 5 (clinical features only/age-only baseline model) had a substantially lower bootstrap mean AUC of 0.6926 (95% CI: 0.6070–0.7695) than the other models incorporating multiple feature types.

The DeLong test further quantified the differences in AUC between models (Table 7). Model 1 significantly outperformed the clinical baseline model (Model 5: ΔAUC = 0.1806, *P* < 0.001), the clinical + blood-indicator model (Model 3: ΔAUC = 0.0607, *P* = 0.011), and the tongue-feature + blood-indicator model (Model 4: ΔAUC = 0.0525, *P* = 0.021). However, the difference in AUC between Model 1 and Model 2 (clinical + tongue features) was not statistically significant (ΔAUC = 0.0306, *P* = 0.187), suggesting that, within the current sample size, the addition of blood indicators increased the AUC numerically but did not provide a statistically significant incremental improvement in discrimination.

**TABLE 7:** Pairwise comparison of AUCs between XGBoost models using the DeLong test.

Model 1	Model 2	AUC_1_	AUC_2_	ΔAUC	P value
Full model	Clinical only (age)	0.8745	0.6939	0.1806	<0.001
Clinical + TCM	Clinical only (age)	0.8439	0.6939	0.15	<0.001
Clinical + blood	Clinical only (age)	0.8138	0.6939	0.1199	0.005
Full model	Clinical + blood	0.8745	0.8138	0.0607	0.011
TCM + blood	Clinical only (age)	0.822	0.6939	0.1281	0.017
Full model	TCM + blood	0.8745	0.822	0.0525	0.021
Full model	Clinical + TCM	0.8745	0.8439	0.0306	0.187
Clinical + TCM	Clinical + blood	0.8439	0.8138	0.0301	0.441
Clinical + TCM	TCM + blood	0.8439	0.822	0.0218	0.525
Clinical + blood	TCM + blood	0.8138	0.822	−0.0083	0.775

Among the three dual-modality models, no statistically significant differences were observed in pairwise comparisons (Model 2 vs. Model 3: *P* = 0.441; Model 2 vs. Model 4: *P* = 0.525; Model 3 vs. Model 4: *P* = 0.775). In contrast, each of Models 2, 3, and 4 significantly outperformed Model 5, indicating that all dual-modality models incorporating tongue features and/or blood indicators provided significantly better discrimination than the age-only baseline model (all *P* < 0.05). However, the differences among the three dual-modality models did not reach statistical significance.

Notably, the bootstrap standard deviation increased monotonically as the number of feature modalities decreased (Model 1: 0.0278 → Model 2: 0.0324 → Model 4: 0.0335 → Model 3: 0.0348 → Model 5: 0.0427), suggesting that reduced feature dimensionality was associated with greater uncertainty in model performance estimates. The bootstrap 95% CIs of the models showed partial overlap between the full-feature and dual-modality models; however, the full-feature model had both a higher point estimate and a higher lower bound of the 95% CI. Overall, Model 1, which integrated clinical, objective tongue, and blood-based features, demonstrated the strongest predictive performance for lung cancer. The DeLong test (Table 7) further confirmed its statistically significant advantage over most dual-modality and baseline models. Model 2, which did not require blood testing, provided a performance-comparable alternative to Model 1 and may therefore have potential clinical utility in resource-limited settings.

### Model explainability

Figure 4A presents a bar plot of the mean absolute SHAP values (Mean |SHAP|) for the full-feature XGBoost model, quantifying the overall contribution of each feature to the model output. The features were ranked in descending order of their contribution as follows: age (0.110), absolute lymphocyte count (LYM#, 0.085), Qi-Deficiency Constitution Score (QDC-Score, 0.083), apolipoprotein C3 (ApoC3, 0.064), tongue coating color L* (TC-L, 0.057), γ-glutamyl transferase (GGT, 0.049), full tongue color b* (FT-B, 0.048), fibronectin (FN, 0.035), Blood-Stasis Constitution Score (BSC-Score, 0.030), qualitative tongue-body color classification (TCC4, 0.029), degree of white coating (TC-White, 0.027), quantitative coating thickness index (CT-Quant, 0.025), and tongue body color L* (TB-L, 0.020).

**FIGURE 4:**
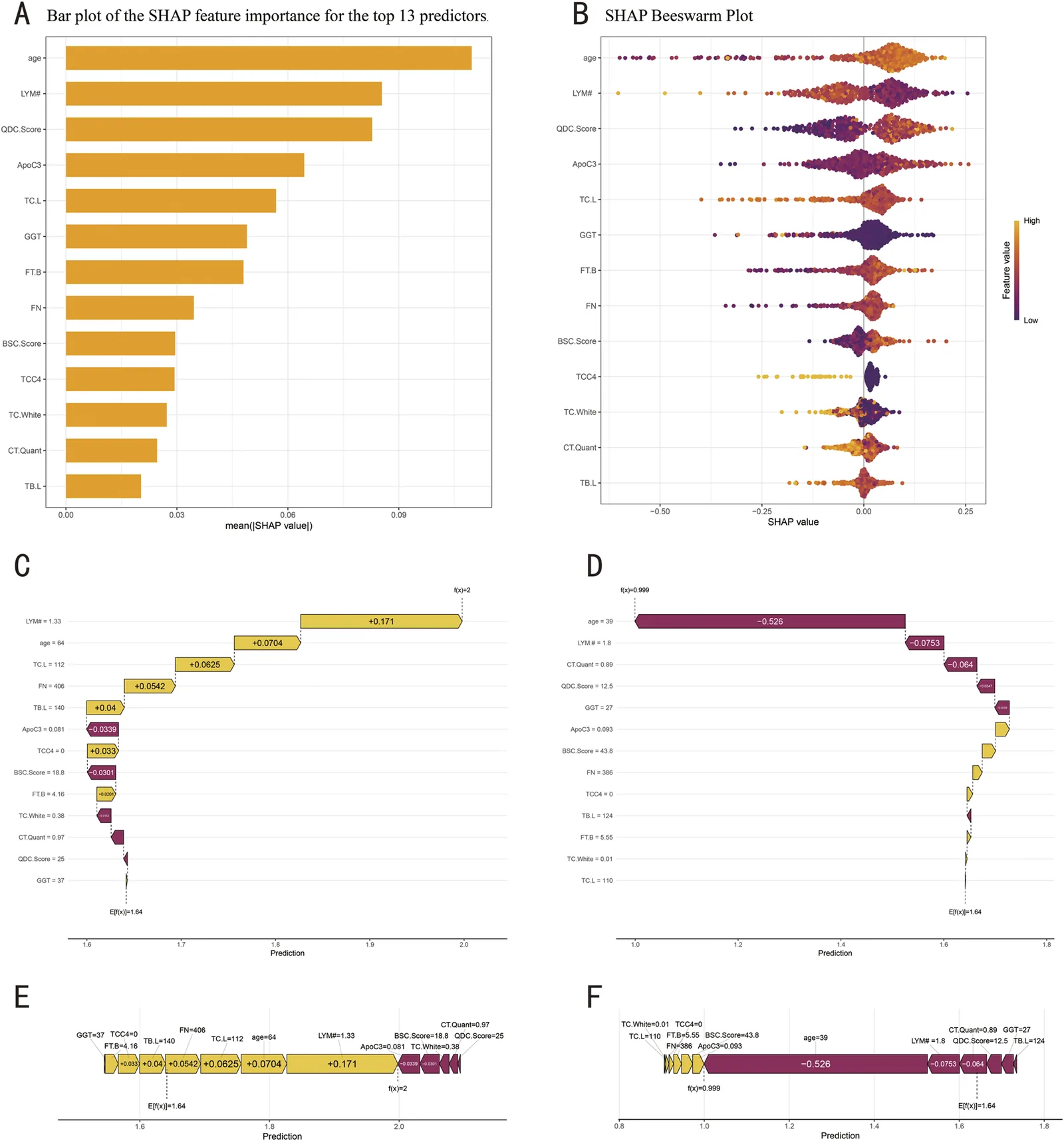
SHAP (Shapley Additive Explanations) analysis for model interpretation. **(A)** Bar plot of the SHAP feature importance for the top 13 predictors. **(B)** Beeswarm plot summarizing the global impact and distribution of SHAP values for each feature. **(C,D)** Individual explanation plots: waterfall plots illustrating the cumulative contribution of features to the model output for **(C)** a representative lung cancer patient and **(D)** a representative non-cancer patient. **(E,F)** Force plots visualizing the local prediction breakdown for **(E)** the same lung cancer patient and **(F)** the same non-cancer patient.

Figure 4B presents the beeswarm plot of SHAP values for individual features in the training set. The color coding represents the magnitude of feature values, with purple indicating low values and yellow/orange indicating high values, thereby illustrating the direction of the associations between feature values and model output.

For age, high values (yellow/orange) were densely distributed in the positive SHAP region on the right, whereas low values (purple) were concentrated in the negative SHAP region on the left, indicating that older age was positively associated with the predicted probability of lung cancer. In contrast, absolute lymphocyte count (LYM#) showed an opposite pattern: high values (yellow) were predominantly located in the negative SHAP region, while low values (purple) tended to cluster in the positive SHAP region, suggesting that lower LYM# was associated with a higher predicted probability of lung cancer.

Among the Traditional Chinese Medicine (TCM) constitution features, high values (yellow/orange) of both the Qi-Deficiency Constitution Score (QDC-Score) and Blood-Stasis Constitution Score (BSC-Score) were densely distributed in the positive SHAP region, whereas low values (purple) were mainly located in the negative SHAP region, suggesting that greater degrees of Qi-deficiency and blood-stasis constitution were associated with a higher predicted probability of lung cancer. Among the laboratory parameters, high values (yellow/orange) of apolipoprotein C3 (ApoC3), γ-glutamyl transferase (GGT), and fibronectin (FN) were predominantly shifted toward the positive SHAP region, indicating that higher levels of these parameters were positively associated with the predicted probability of lung cancer.

For the tongue-image features, high values (yellow/orange) of tongue coating color L* (TC-L), degree of white coating (TC-White), and quantitative coating thickness index (CT-Quant) were predominantly associated with negative SHAP values, whereas low values (purple) were associated with positive SHAP values. In contrast, high values (yellow/orange) of full tongue color b* (FT-B) were mainly associated with positive SHAP values, while low values (purple) were associated with negative SHAP values. For tongue body color L* (TB-L) and the categorical qualitative tongue color classification (TCC4), which represents pathological tongue colors such as purple or deep-red/crimson tongue, higher encoded values were distributed across both positive and negative SHAP regions or predominantly in the negative SHAP region, whereas lower encoded values were concentrated near the neutral SHAP region. These findings suggest that deviations in tongue-body lightness and tongue-body color from the normal range may be closely associated with an increased predicted risk of lung cancer.

Overall, the beeswarm plot illustrates the direction in which individual features contribute to the XGBoost model’s predictions. With the exception of LYM#, which showed an inverse association, the remaining features generally demonstrated a pattern in which higher feature values were associated with a higher predicted probability of lung cancer. Together with the ranking based on mean absolute SHAP values, these findings highlight the complementary discriminative contributions of multimodal features to lung cancer identification.

Figures 4C,D are waterfall plots corresponding to individual lung cancer and non-tumor patients, where each bar represents a feature; red indicates a negative contribution to the prediction (risk reduction), yellow indicates a positive contribution (risk increase), and the size of the arrow reflects the magnitude of the feature contribution. Figures 4E,F are force plots showing how 13 feature values add to or subtract from the baseline prediction E[f(x)], according to their contributions, to produce the final prediction f(x).

Figures 4C,E correspond to the same lung cancer patient. In contrast, Figures 4D,F depict feature contributions for the corresponding non-tumor patient.

## Discussion

Lung cancer is a disease that severely compromises patient health and imposes a substantial burden on both individuals and society. Owing to its insidious onset and the insufficient implementation of lung cancer screening worldwide, particularly in developing countries, the disease is often detected at an advanced stage, resulting in poor patient prognosis. Our study is the first multimodal analysis that integrates Traditional Chinese Medicine (TCM) tongue diagnosis, constitution scores, pulse assessment, and routine blood indicators in lung cancer and non-tumor patients; we identified 13key features and developed a Random Forest (RF) model to predict lung cancer, which demonstrated strong discriminative performance. The features used in this model are easy to obtain, convenient, and low-cost, while maintaining accuracy, making it highly suitable for large-scale lung cancer screening. Machine learning (ML) has shown increasing value in disease prediction and clinical risk stratification.

Growing evidence indicates that ML-based models outperform traditional statistical approaches in multiple diagnostic contexts. For example, the sensitivity of convolutional neural networks (CNNs) for detecting pulmonary nodules on chest CT images has surpassed radiologists in some settings (Thanoon et al., 2023; Shatnawi, 2025). Gradient boosting models (e.g., XGBoost) applied to comprehensive emergency triage data substantially improve the accuracy of early sepsis screening (Liu et al., 2025). These studies collectively highlight the significant potential of ML to improve diagnostic accuracy and facilitate early disease detection. In our study, the integration of multimodal features—including tongue and pulse characteristics, TCM constitution scores, and routine blood parameters—enabled strong discriminative performance. Among the six evaluated models, XGBoost (eXtreme Gradient Boosting) achieved the best overall performance across key evaluation metrics, including the area under the receiver operating characteristic curve (AUC) and Youden index, establishing it as the optimal predictive model in this study. Although the DeLong test showed no statistically significant difference in AUC between XGBoost and Random Forest (P = 0.832), the two models exhibited subtly different clinical trade-offs for lung cancer screening. Random Forest achieved modestly higher sensitivity (0.8590 vs. 0.8205), which may be advantageous for minimizing missed cases in population-based primary screening. Conversely, XGBoost demonstrated slightly higher specificity (0.6842 vs. 0.6711) and a superior Youden index (0.6066 vs. 0.5975), indicating a more balanced overall discriminative performance. Given that these differences were small and statistically non-significant, the choice between the two models in clinical practice may depend less on differences in discrimination than on operational considerations such as computational efficiency, interpretability, and workflow integration. Nevertheless, XGBoost demonstrated the highest AUC and Youden index among all evaluated classifiers and was therefore selected as the preferred model for general lung cancer risk stratification, whereas Random Forest may represent an acceptable alternative when marginally higher sensitivity is prioritized.

The favorable performance of XGBoost in this study may be partly explained by its ability to model complex and nonlinear relationships among heterogeneous clinical features. XGBoost employs a gradient-boosting framework that constructs weak learners, typically decision trees, sequentially in an additive manner, with each newly added tree focusing on correcting the residual errors of the preceding ensemble. This iterative process enables the model to progressively capture complex feature patterns and interactions. XGBoost also incorporates L1/L2 regularization and a shrinkage parameter (learning rate) to constrain model complexity and reduce the risk of overfitting during iterative optimization. In addition, its regularization strategy and column subsampling (feature subsampling) can help control model variance and improve robustness to noise and heterogeneous data (Zhang et al., 2019). These characteristics are particularly relevant to the present study, which integrates multimodal clinical information, including routine laboratory parameters, TCM constitution scores, and quantitative tongue-image features, within a moderate-sized clinical dataset.

Beyond predictive performance, XGBoost also offers practical advantages for model interpretation. It provides built-in feature-importance measures and is highly compatible with explainable artificial intelligence frameworks such as SHAP. This combination of predictive flexibility and interpretability enabled us not only to identify the best-performing classifier but also to further characterize the relative contributions and directions of individual features within the final model. Accordingly, the subsequent SHAP analysis provided an interpretable framework for examining how demographic, laboratory, TCM constitution, and tongue-image features contributed to lung cancer prediction, thereby offering a more transparent basis for potential clinical decision support.

Using the LASSO algorithm, 13 robust predictors of lung cancer were selected, and SHAP analysis quantified their influence on model predictions: Age ranked first, followed by laboratory parameters (LYM#, ApoC3, GGT, and FN) and TCM constitution and tongue-image features (QDC-Score, BSC-Score, TCC4, TC-White, FT-B, TC-L, TB-L, and CT-Quant). The SHAP summary and beeswarm plots indicated that advanced age was associated with a persistent rise in lung cancer prediction, while hematologic and TCM-derived features displayed divergent directions within the cohort. Age is the major epidemiological risk factor for lung cancer: multiple population studies show that incidence rises sharply after 60–65 years, reflecting cumulative carcinogen exposure, accumulation of somatic mutations, and age-related immune senescence (Sharma, 2022). This is consistent with our SHAP findings: higher age corresponded to the largest positive SHAP contribution.

To assess whether the model performance was primarily attributable to age, we compared XGBoost models incorporating different combinations of features (Table 6). The model based on age alone showed only moderate discriminatory ability (AUC = 0.6939, 95% CI: 0.6112–0.7766), which was substantially lower than that of the full-feature model (AUC = 0.8745, 95% CI: 0.8219–0.9271). Adding TCM-derived features, including tongue-image features and constitution scores, to age increased the AUC to 0.8439, corresponding to an incremental gain of 0.1500. In contrast, adding routine blood parameters to age increased the AUC to 0.8138, with an incremental gain of 0.1199. When all three feature domains were combined, the full model achieved an AUC of 0.8745, representing an improvement of 0.1806 over the age-only model. Furthermore, adding blood parameters to the Age + TCM model provided a marginal gain of 0.0306, whereas adding TCM-derived features to the Age + Blood model resulted in a marginal gain of 0.0607.

These findings demonstrate that although age represents an important baseline predictor, it does not account for the overall discriminative performance of the model. Both TCM-derived features and routine blood parameters provide substantial complementary information beyond age, and their integration into a multimodal feature set significantly improves the discriminatory ability of the model for lung cancer identification.

Importantly, these findings also support the continued interpretation of the other variables retained by LASSO. Although some of these features had smaller individual contributions than age according to SHAP analysis, their collective inclusion contributed to the incremental predictive value of the multimodal model. Therefore, the clinical and biological relevance of the LASSO-selected features should not be assessed solely according to their individual SHAP rankings, but rather in the context of their complementary contributions to model discrimination.Among the blood-related variables selected by LASSO, several require particular attention. First, absolute lymphocyte count (LYM#) exhibited a negative association in SHAP: lower LYM# values were linked to a higher predicted probability of lung cancer. From a biological standpoint, lymphocyte depletion reflects compromised cell-mediated immunity and weakened antitumor surveillance; numerous studies (Li et al., 2023; Ray-Coquard et al., 2010) report that lower lymphocyte counts at baseline or diagnosis correlate with adverse cancer prognosis. Our results are consistent with this literature.Second, apolipoprotein C3 (ApoC3) was included in the model, and higher values corresponded to a greater probability of lung cancer. Increasing evidence supports a role for APOC3 in cancer: in oral squamous cell carcinoma (OSCC), APOC3 binds ENO1 to induce IL-8 secretion, activating STAT3 signaling and suppressing CD8^+^ T-cell proliferation, thereby facilitating immune escape (Wang et al., 2025). In hepatocellular carcinoma (HCC), high APOC3 expression is associated with shorter overall survival (OS), suggesting that APOC3 may promote tumor progression and deterioration (Wang, 2025). Although Emerging preclinical and small-sample clinical data suggest a potential association between elevated ApoC3 and malignant tumor progression in oral squamous cell carcinoma and hepatocellular carcinoma. Direct large-scale epidemiological evidence verifying the causal relationship between serum ApoC3 and lung cancer incidence remains insufficient, so this study only reports an observational correlational trend without inferring definite causality. Third, GGT is commonly positively associated with multiple cancers in epidemiological studies, especially hepatobiliary and gastrointestinal tumors, and may relate to oxidative stress and metabolic dysregulation (Ramandi et al., 2025; Van Hemelrijck et al., 2011). However, in our dataset, higher GGT levels were associated with a lower predicted risk of lung cancer. Two non-exclusive explanations may account for this: first, the real-world control group contained many patients with inflammatory gastrointestinal and hepatobiliary conditions (e.g., cholecystitis, pancreatitis, chronic hepatobiliary disease) (Ramandi et al., 2025), which themselves markedly elevate GGT, shifting the control distribution upward and creating an apparent negative association between GGT and lung cancer. On the other hand, meta-analyses indicate that the relationship between GGT and cancer is site-specific (stronger for liver and colorectal malignancies), suggesting that the inverse coefficient observed here likely reflects organ-specific heterogeneity or residual confounding rather than a protective biological effect of GGT on lung cancer. Fourth, Fibronectin (FN) showed a positive association in the SHAP analysis, with higher FN levels corresponding to a higher predicted probability of lung cancer. This finding is biologically plausible given the established role of FN as a major structural glycoprotein of the extracellular matrix (ECM), where it participates in tumor microenvironment remodeling, cell–matrix adhesion, and cellular migration. In lung cancer, tumor-associated fibroblasts (CAFs) can produce abundant FN, which may promote tumor cell invasion and migration through integrin-mediated activation of downstream signaling pathways, including the FAK/Src and PI3K–AKT axes. FN has also been implicated in epithelial–mesenchymal transition (EMT), a process closely associated with tumor invasion and metastatic progression. Previous studies have reported increased FN expression in non-small cell lung cancer (NSCLC), supporting its potential involvement in tumor progression. In addition, circulating FN levels have been reported to be elevated in patients with several solid malignancies, including lung cancer, potentially reflecting systemic responses to tumor-associated tissue injury, chronic inflammation, and extracellular matrix remodeling.

Although prospective evidence from large-scale studies directly evaluating the association between circulating FN levels and lung cancer risk remains limited, existing molecular and clinicopathological evidence supports a potential role for FN in the tumor-associated microenvironment and malignant progression. Our findings are broadly consistent with these observations, suggesting that FN may represent not only an important component of the tumor microenvironment but also a potential peripheral blood biomarker contributing to lung cancer risk stratification. Nevertheless, the positive association identified by SHAP analysis should be interpreted as a model-based association rather than evidence of a causal relationship, and further prospective and mechanistic studies are warranted to determine whether circulating FN has independent value for lung cancer risk prediction.

Among the 13 important features selected by LASSO, several indicators directly related to TCM syndromes/constitutional types showed marked contributions. First, both the Blood Stasis Constitution Score (BSC-Score) and Qi Deficiency Constitution Score (QDC-Score),showed positive SHAP contributions, indicating that higher scores correspond to a greater predicted risk of lung cancer. This finding is consistent with prior clinical studies and meta-analyses: lung cancer patients more frequently exhibit syndromes such as Qi-deficiency and blood stasis, which are also more common in disease progression (Yu et al., 2022). According to TCM theory, Qi deficiency indicates insufficient vital Qi and weakened resistance; blood stasis corresponds to microcirculatory impairment and local hypoxia. These pathomechanisms exhibit conceptual concordance with the pathological processes of tumor initiation and progression (Yu et al., 2022). Regarding tongue-image indicators, we found that lower CT-Quant (quantitative coating thickness/area) values were associated with higher lung cancer risk, meaning that the lung cancer group tended to have reduced or easily exfoliated tongue coating. This observation is consistent with prior tongue-diagnosis studies (Shi et al., 2023a), where progressive or advanced diseases often show thinning or peeling of tongue coating; in TCM, such features are commonly associated with Qi-deficiency or Yin-deficiency constitutions. Qi-deficiency results in diminished stomach Qi incapable of nourishing the tongue, producing coating exfoliation; impaired fluid ascension similarly contributes to coating loss. This matches the elevated QDC-Score observed in our model.

Second, degree of white coating (TC-White) showed a similar pattern, with greater white-coating coverage (yellow/orange points) corresponding to negative SHAP values, whereas reduced white coating (purple points) was associated with positive SHAP values. Together with the findings for quantitative coating thickness index (CT-Quant), these results suggest that a reduced amount of tongue coating, characterized by a thin or scant coating rather than a thick or greasy coating, may represent a tongue feature associated with a higher predicted probability of lung cancer. Tongue coating color L* (TC-L) showed a consistent directional pattern: lower lightness values, indicating a darker coating (purple points), were associated with positive SHAP values, whereas higher lightness values, indicating a lighter coating (yellow/orange points), were associated with negative SHAP values. Reduced tongue-coating lightness and a darker or duller coating color may be traditionally associated with patterns such as blood stasis or phlegm-dampness accumulation, which is broadly consistent with the positive contribution of the Blood-Stasis Constitution Score (BSC-Score) observed in the model.

In contrast to the aforementioned tongue-coating features, full tongue color b* (FT-B) exhibited a positive association with the model output. According to the CIE-Lab color space, FT-B represents the blue–yellow axis (b* axis), with higher values indicating a more yellowish tongue color and lower values indicating a more bluish color. The beeswarm plot showed that high FT-B values (yellowish tones; yellow/orange points) were associated with positive SHAP values, whereas low FT-B values (bluish tones; purple points) were associated with negative SHAP values. This pattern suggests that a more yellowish tongue color was associated with a higher predicted probability of lung cancer, whereas a more bluish tongue color was associated with a lower predicted probability. From a TCM perspective, a yellowish tongue color is commonly associated with patterns such as damp-heat accumulation or phlegm-heat retention and may reflect systemic inflammatory or metabolic alterations associated with the tumor milieu. However, these traditional interpretations should be regarded as potential explanatory frameworks rather than direct evidence of specific underlying biological mechanisms.

The SHAP distributions of tongue body color L* (TB-L) and qualitative tongue color classification (TCC) were more complex and suggested potential nonlinear associations. For TB-L, high lightness values were distributed across both positive and negative regions of the SHAP axis, whereas low lightness values were concentrated closer to the zero line, suggesting that the association between tongue-body lightness and the predicted probability of lung cancer may not follow a simple linear pattern. For TCC, a categorical feature, TCC4 represents a purple pathological tongue color. Higher encoded values were predominantly distributed in the negative SHAP region, whereas lower encoded values were concentrated around the neutral region. This pattern was directionally similar to that observed for FT-B, suggesting that specific tongue-body color characteristics, including purple discoloration, may be associated with a lower model-predicted probability of lung cancer. Nevertheless, because TCC is a categorical variable represented by numerical encoding, the magnitude and direction of its encoded values should not be interpreted as a continuous biological gradient.

Previous clinical studies have reported that a bluish-purple tongue color is commonly associated with blood stasis, impaired microcirculatory perfusion, and certain tumor-related conditions, and may occur more frequently in patients with advanced malignancies, including those with elevated platelet counts and coagulation-related biomarkers (Zeng et al., 2024). The apparent discrepancy between these observations and the present findings, particularly the negative SHAP contribution observed for some categorical or quantitative tongue-color features, may have several plausible explanations.

First, the real-world control group in our study may have included individuals with non-malignant conditions capable of producing pronounced or atypical tongue features, such as trauma, severe blood-stasis-related conditions, or localized inflammatory disorders. The relatively high frequency of purple or markedly pale tongue characteristics among these controls could have weakened or even reversed the apparent association between these tongue features and lung cancer classification. Second, the tongue-color characteristics identified in previous studies may reflect differences in disease stage and clinical context. For example, bluish-purple tongue discoloration may be more closely associated with advanced disease, impaired circulation, or systemic coagulation abnormalities, whereas our study primarily addressed the discrimination of lung cancer from non-cancer controls rather than the characterization of tumor stage or progression. Third, prominent white tongue coating among controls may reflect benign or transient conditions, including fungal colonization, local inflammation, or reversible coating thickening, rather than tumor-associated physiological changes. Consequently, categorical descriptions of tongue coating or color may capture heterogeneous underlying conditions, whereas quantitative features such as reduced coating thickness, lower coating lightness, or specific tongue-body color parameters may provide more refined discriminatory information.

The mTOR signaling pathway acts as a core hub linking aging, metabolism, inflammation and anti-tumor immunity, which provides an upstream molecular framework to interpret the predictive value of our screened multimodal markers. Hyperactivated mTOR promotes cellular senescence with age accumulation, drives lipid metabolic reprogramming and ApoC3 overexpression, amplifies chronic pulmonary inflammatory response and oxidative stress, and inhibits lymphocyte anti-tumor function by suppressing T cell activation (Fan et al., 2022; Fan et al., 2026). In lung epithelial cells, sustained mTOR overactivation accelerates malignant transformation and promotes microcirculation disturbance corresponding to blood-stasis TCM tongue manifestations (Fan and Xu, 2026). Meanwhile, mTOR hyperactivity aggravates systemic low-grade inflammation, matching the high predictive weight of damp-heat and Qi-deficiency constitution scores (Fan et al., 2019). The combined alterations of age, blood lipid, immune and TCM tongue/constitution markers observed in our model can be partially explained by dysregulated mTOR signaling upstream of tumorigenesis.Taken together, the apparent inconsistency between qualitative and quantitative tongue features may therefore be attributable, at least in part, to differences in study design, control-group composition, disease stage, and feature representation. Rather than indicating that the established clinical associations of bluish-purple tongue coloration are necessarily contradictory to our findings, the present results suggest that the predictive value of tongue characteristics may be context-dependent and influenced by how these features are defined and quantified. Further prospective studies with well-characterized control populations, detailed disease-stage information, and standardized quantitative tongue assessment are warranted to clarify the clinical significance of these tongue features in lung cancer risk stratification.

This low-cost multimodal prediction model possesses prominent translational value for primary community health service and tiered medical systems, and we propose a complete real-world deployment pathway as follows: (1) Hardware terminal integration: Embed the optimized XGBoost prediction algorithm into portable integrated tongue diagnosis devices with built-in blood routine data input modules and TCM constitution questionnaire mini-programs. Community doctors can complete one-stop collection of tongue images, basic blood test results and constitution scores within 5 min without high-end imaging equipment. (2) Mobile auxiliary application: Develop a matched mobile phone App for physical examination crowds. Users can finish online TCM constitution scoring independently, upload standardized tongue photos taken by mobile cameras, and upload outpatient blood test reports to obtain instant lung cancer risk prediction results. (3) Tiered screening workflow: The model acts as a first-line risk stratification tool in community health centers and physical examination clinics. Individuals with high predicted risk output are directly referred to tertiary hospitals for chest CT and pathological confirmation; low-risk individuals receive routine annual follow-up without unnecessary CT radiation exposure. (4) Public health advantages: Compared with universal CT population screening, this model greatly reduces overall screening expenditure and radiation burden, especially applicable for rural and underdeveloped regions with limited medical resources. Combined with standardized objective TCM diagnostic equipment, this multimodal tool can popularize standardized TCM tumor early screening in primary medical institutions and balance uneven medical resource distribution between urban and rural areas.

In this study, a lung cancer screening model was developed using hematological indicators, tongue features, and constitution scores (TCM). The model demonstrated good accuracy in identifying high-risk individuals among the non-tumor population, and the assessment process was simple, low-cost, and imposed only minimal burden on participants. Combining TCM characteristics with biomedical variables revealed synergistic benefits and may inform the development of multidimensional diagnostic systems. However, this study also has limitations. First, the retrospective design restricted the sample size of non-tumor participants because the inclusion criteria required the availability of both comprehensive TCM data and complete conventional clinical laboratory parameters. Prospective, multicenter cohort studies are needed to validate and update the model in the future. Second, the completion rate of the constitution questionnaire was low, possibly due to the number of items. Based on our results, the questionnaire may be streamlined to improve clinical feasibility. Third, pulse diagnosis indicators did not enter the final model, possibly due to insufficient acquisition accuracy, immature feature extraction methods, operator variability, and the non-specificity of pulse features. Moreover, the single-center sample may affect external generalizability, and some variables may still be influenced by medications, comorbidities, or lifestyle factors. Although SHAP analysis improved model interpretability, deeper analyses combining pathological mechanisms and clinical evidence are still required. Overall, this study proposed a low-cost and easily accessible lung cancer screening strategy, and prospective research remains essential for model optimization and clinical translation.

Several limitations should be emphasized. First, the model lacks independent external validation, and its generalizability to other hospitals or community populations remains unconfirmed. In addition, the relatively small validation sample size (n = 156) may have limited the precision and reliability of comprehensive calibration estimates, particularly for calibration metrics requiring sufficient sample size across the range of predicted probabilities. More robust calibration assessment, including evaluation of calibration-in-the-large, calibration slope, and other calibration metrics, will therefore be prioritized in future independent, external, multicenter validation cohorts. Second, smoking history was excluded because of a high missing rate (38.5%) in retrospective outpatient records, which may cause residual confounding. Third, non-tumor controls were confirmed cancer-free primarily by clinical history and chest CT without systematic pathology or long-term follow-up, and they represent a symptomatic outpatient population rather than an asymptomatic screening cohort, potentially limiting applicability to population-based screening. Fourth, detailed treatment history, comorbidities, and medication records were incompletely documented for lung cancer patients. Fifth, this model is not intended to replace low-dose CT; it should serve as a low-cost, non-radiative pre-screening or triage tool, with high-risk individuals referred for confirmatory LDCT. Future prospective multicenter studies in independent, community-based cohorts are warranted before clinical deployment.

## Conclusion

In this study, we developed a lung cancer screening model based on easily accessible clinical indicators, including routine laboratory parameters, tongue features, and constitution scores (TCM). Although the model is based on routine, low-cost clinical variables, it achieved high accuracy in distinguishing lung cancer patients from non-tumor individuals. Our findings suggest that integrating TCM-related features with biomedical parameters may provide complementary diagnostic value and offer a novel and economical strategy for early lung cancer risk stratification, especially in settings where access to advanced imaging or invasive procedures is limited.

## Data Availability

The original contributions presented in the study are included in the article/Supplementary Material, further inquiries can be directed to the corresponding authors.

## References

[B1] Aredo J. V. Wakelee H. A. (2024). Top advances of the year: perioperative therapy for lung cancer. Cancer 130 (17), 2897–2903. 10.1002/cncr.35357 38717993

[B2] Cao A. He H. Jing M. Yu B. Zhou X. (2017). Shenfu injection adjunct with platinum-based chemotherapy for the treatment of advanced non-small-cell lung cancer: a meta-analysis and systematic review. Evid. Based Complement. Altern. Med. 2017, 1068751. 10.1155/2017/1068751 29234363 PMC5688370

[B3] Cao Y. Li P. Zhu Y. Wang Z. Tang N. Li Z. (2025). Artificial intelligence-enabled novel atrial fibrillation diagnosis system using 3D pulse perception flexible pressure sensor array. ACS Sens. 10 (1), 272–282. 10.1021/acssensors.4c02395 39757849

[B4] Chen L. C. Papandreou G. Kokkinos I. Murphy K. Yuille A. L. (2018). DeepLab: semantic image segmentation with deep convolutional nets, atrous convolution, and fully connected CRFs. IEEE Trans. Pattern Analysis Mach. Intell. 40 (4), 834–848. 10.1109/TPAMI.2017.2699184 28463186

[B5] Chen J. Yang J. Qin Y. Sun C. Xu J. Zhou X. (2022). Tongue features of patients with granulomatous lobular mastitis. Med. Baltim. 101 (46), e31327. 10.1097/MD.0000000000031327 36401439 PMC9678557

[B6] Chen J. Sun Y. Li J. Lyu M. Yuan L. (2024). In-depth metaproteomics analysis of tongue coating for gastric cancer: a multicenter diagnostic research study. Microbiome 12 (1), 6. 10.1186/s40168-023-01730-8 38191439 PMC10773145

[B7] Crosby D. Bhatia S. Brindle K. M. Coussens L. M. Dive C. Emberton M. (2022). Early detection of cancer. Science 375 (6586), eaay9040. 10.1126/science.aay9040 35298272

[B8] Duan B. Xie J. Rui Q. Zhang W. Xi Z. (2018). Effects of Shengmai injection add-on therapy to chemotherapy in patients with non-small cell lung cancer: a meta-analysis. Support Care Cancer 26 (7), 2103–2111. 10.1007/s00520-018-4167-4 29594484

[B9] Fan J. Xu Y. (2026). Molecular mechanisms underlying the lifespan and healthspan benefits of dietary restriction across species. Front. Genet. 17, 1771707. 10.3389/fgene.2026.1771707 41695773 PMC12900492

[B10] Fan J. Gao Y. Lu Y. Wu W. Yuan S. Wu H. (2019). PKAc-directed interaction and phosphorylation of Ptc is required for Hh signaling inhibition in Drosophila. Cell Discov. 5, 44. 10.1038/s41421-019-0112-z 31636957 PMC6796939

[B11] Fan J. Yuan Z. Burley S. K. Libutti S. K. Zheng X. F. S. (2022). Amino acids control blood glucose levels through mTOR signaling. Eur. J. Cell Biol. 101 (3), 151240. 10.1016/j.ejcb.2022.151240 35623230 PMC10035058

[B12] Fan J. Zhang X. Zhang J. Zhao T. Burley S. K. Zheng X. F. S. (2026). PDX1 phosphorylation at S61 by mTORC1 links nutrient signaling to β cell function and metabolic disease. Cell Rep. 45 (1), 116811. 10.1016/j.celrep.2025.116811 41528843 PMC12949489

[B13] Fei Z. (2012). Mai zhen yi de yan jiu xian zhuang ji dui cun kou san bu mai xiang ke guan jian ce de chu bu she xiang. Shanghai Zhongyiyao Daxue Xuebao 26 (4), 7–10. 10.16306/j.1008-861x.2012.04.010

[B14] He X. McLean J. S. Edlund A. Yooseph S. Hall A. P. Liu S. Y. (2015). Cultivation of a human-associated TM7 phylotype reveals a reduced genome and epibiotic parasitic lifestyle. Proc. Natl. Acad. Sci. U. S. A. 112 (1), 244–249. 10.1073/pnas.1419038112 25535390 PMC4291631

[B15] He K. Zhang X. Ren S. Sun J. (2016). “Deep residual learning for image recognition,” in 2016 IEEE Conference on Computer Vision and Pattern Recognition (CVPR), 770–778. 10.1109/CVPR.2016.90

[B16] Iwendi C. Bashir A. K. Peshkar A. Sujatha R. Chatterjee J. M. Pasupuleti S. (2020). COVID-19 patient health prediction using boosted random Forest algorithm. Front. Public Health 8, 357. 10.3389/fpubh.2020.00357 32719767 PMC7350612

[B17] Ke G. Meng Q. Finley T. (2017). “LightGBM: a highly efficient gradient boosting decision tree,” in Neural Information Processing Systems. Available online at: https://api.semanticscholar.org/CorpusID:3815895 (Accessed September 10, 2026).

[B18] Li S. Yao W. Liu R. Lu Y. Zhang H. Liang X. (2023). Severe lymphopenia as a prognostic factor in rectal cancer patients receiving adjuvant chemoradiotherapy: a retrospective study. Sci. Rep. 13 (1), 7566. 10.1038/s41598-023-34145-4 37161043 PMC10170099

[B19] Lim J. Li J. Feng X. (2025). Machine Learning Classification of Polycystic Ovary Syndrome Based on Radial Pulse Wave Analysis. 10.1186/s12906-023-04249-5 PMC10644435 37957660

[B20] Lin S. C. A. Chu P. Y. Chen L. L. Su Y. C. Wang S. M. (2017). The prevalence rate of deviations in body constitutions and related factors in follow-up stage breast cancer patients—A nationwide study. Complementary Ther. Med. 32, 49–55. 10.1016/j.ctim.2017.03.013 28619304

[B21] Little R. J. A. (2014). Statistical Analysis with Missing Data. 2nd ed. Incorporated: John Wiley & Sons.

[B22] Liu Z. Shu W. Li T. Zhang X. Chong W. (2025). Interpretable machine learning for predicting sepsis risk in emergency triage patients. Sci. Rep. 15 (1), 887. 10.1038/s41598-025-85121-z 39762406 PMC11704257

[B23] Ning D. Jin M. Xv T. Sun J. Li M. (2018). Homoisoflavanone-1 isolated from polygonatum odoratum arrests the cell cycle and induces apoptosis in A549 cells. Oncol. Lett. 16, 3545–3554. 10.3892/ol.2018.9085 30127960 PMC6096101

[B24] Ogunleye A. Wang Q. G. (2020). XGBoost model for chronic kidney disease diagnosis. IEEE/ACM Trans. Comput. Biol. Bioinform 17 (6), 2131–2140. 10.1109/TCBB.2019.2911071 30998478

[B25] Potter A. L. Rosenstein A. L. Kiang M. V. Shah S. A. Gaissert H. A. Chang D. C. (2022). Association of computed tomography screening with lung cancer stage shift and survival in the United States: quasi-experimental study. BMJ 376, e069008. 10.1136/bmj-2021-069008 35354556 PMC8965744

[B26] Ramandi A. George J. Behnoush A. H. (2025). The Association Between Serum Gamma‐Glutamyl Transferase and Gastrointestinal Cancer Risk: A Systematic Review and Meta‐Analysis. 10.1002/cam4.70581 PMC11736428 39817495

[B27] Ray-Coquard I. Cropet C. Glabbeke M. V. (2010). Lymphopenia As A Prognostic Factor For Overall Survival In Advanced Carcinomas, Sarcomas And Lymphomas. Published online. 10.1158/0008-5472.CAN-08-3845 PMC2775079 19549917

[B28] Sharma R. (2022). Mapping of global, regional and national incidence, mortality and mortality-to-incidence ratio of lung cancer in 2020 and 2050. Int. J. Clin. Oncol. 27 (4), 665–675. 10.1007/s10147-021-02108-2 35020103 PMC8753949

[B29] Shatnawi M. Q. (2025). Deep Learning-based Approach to Diagnose Lung Cancer Using CT-scan Images. Published online.

[B30] Shi Y. Guo D. Chun Y. Liu J. Liu L. Tu L. (2023a). A lung cancer risk warning model based on tongue images. Front. Physiol. 14, 1154294. 10.3389/fphys.2023.1154294 37324390 PMC10267397

[B31] Shi Y. Wang H. Yao X. Li J. Liu J. Chen Y. (2023b). Machine learning prediction models for different stages of non-small cell lung cancer based on tongue and tumor marker: a pilot study. BMC Med. Inf. Decis. Mak. 23 (1), 197. 10.1186/s12911-023-02266-5 37773123 PMC10542664

[B32] Siegel R. L. Kratzer T. B. Giaquinto A. N. Sung H. Jemal A. (2025). Cancer statistics, 2025. CA A Cancer J. Clin. 75 (1), 10–45. 10.3322/caac.21871 39817679 PMC11745215

[B33] Sterne J. A. C. White I. R. Carlin J. B. Spratt M. Royston P. Kenward M. G. (2009). Multiple imputation for missing data in epidemiological and clinical research: potential and pitfalls. BMJ 338, b2393. 10.1136/bmj.b2393 19564179 PMC2714692

[B34] Su Y. C. Chen L. L. Lin J. D. Lin J. S. Huang Y. chia Lai J. S. (2008). BCQ+: a body constitution questionnaire to assess Yang-Xu. Complement. Med. Res. 15 (6), 327–334. 10.1159/000175938 19142042

[B35] Thanoon M. A. Zulkifley M. A. Mohd Zainuri M. A. A. Abdani S. R. (2023). A review of deep learning techniques for lung cancer screening and diagnosis based on CT images. Diagnostics 13 (16), 2617. 10.3390/diagnostics13162617 37627876 PMC10453592

[B36] Van Hemelrijck M. Jassem W. Walldius G. Fentiman I. S. Hammar N. Lambe M. (2011). Gamma-glutamyltransferase and risk of cancer in a cohort of 545,460 persons – the Swedish AMORIS study. Eur. J. Cancer 47 (13), 2033–2041. 10.1016/j.ejca.2011.03.010 21486691

[B37] Wang X. (2025). Diagnostic and Prognostic Significance of mRNA Expressions of Apolipoprotein A and C Family Genes in Hepatitis B Virus‐Related Hepatocellular Carcinoma. 10.1002/jcb.29131 31211449

[B38] Wang Q. Jin Q. (2012). Ya Jiankang Zhongyi Tizhi Bian Shi Yu Tiao Li. China Press of Traditional Chinese Medicine.

[B39] Wang J. Man Q. Zhong N. (2025). ENO1 Binds to ApoC3 and Impairs the Proliferation of T Cells via IL-8/STAT3 Pathway in OSCC. 10.3390/ijms232112777 PMC9654972 36361568

[B40] Wang X. Luo S. Tian G. Rao X. He B. Sun F. (2022). Deep learning based tongue prickles detection in traditional Chinese medicine. Evid. Based Complement. Altern. Med. 2022, 5899975. 10.1155/2022/5899975 36185091 PMC9522517

[B41] Yu X. Yan L. Lan Q. Wan L. Xiong J. Zhang L. (2022). Correlation between TCM constitutional types and lung carcinoma in various geographical areas: a systematic review and meta-analysis. Contrast Media Mol. Imaging 2022, 5660231. 10.1155/2022/5660231 36072639 PMC9402362

[B42] Yuan L. Yang L. Zhang S. Xu Z. Qin J. Shi Y. (2023). Development of a tongue image-based machine learning tool for the diagnosis of gastric cancer: a prospective multicentre clinical cohort study. eClinicalMedicine 57, 101834. 10.1016/j.eclinm.2023.101834 36825238 PMC9941057

[B43] Zeng L. Z. Cui J. Jiang T. Liu H. D. Gong Y. B. (2024). Study on the difference and regularity of tongue images in 309 patients with different pathological stages of non-small cell lung cancer. THC 32 (3), 1403–1420. 10.3233/THC-230372 38043028 PMC11091635

[B44] Zhang Z. Zhao Y. Canes A. Steinberg D. Lyashevska O. (2019). Written on behalf of AME Big-Data Clinical Trial Collaborative Group. Predictive analytics with gradient boosting in clinical medicine. Ann. Transl. Med. 7 (7), 152. 10.21037/atm.2019.03.29 31157273 PMC6511546

[B45] Zhang G. He X. Li D. Tian C. Wei B. (2022). Automated screening of COVID-19-Based tongue image on Chinese medicine. Biomed. Res. Int. 2022, 6825576. 10.1155/2022/6825576 35782081 PMC9246631

