# Supplementary material for: A preliminary study of a machine-learning prediction model for lung cancer diagnosis based on routine blood indicators and traditional chinese medicine diagnostic parameters
Source: Front Pharmacol. 2026 Sep 22;17:1916858. doi: 10.3389/fphar.2026.1916858 (PMC13638356; doi:10.3389/fphar.2026.1916858)
Supplement: Supplementary file 1 [file Table1.docx]

# Supplementary Table 1. Specific diagnoses of the 205 non-cancer controls.

| Category | Specific Diagnosis | n | Percentage (%)* |
| --- | --- | --- | --- |
| Hepatobiliary diseases | Cholecystitis | 73 | 35.6 |
| (n = 114, 55.6%) | Gallstones | 20 | 9.8 |
|  | Gallbladder polyp | 8 | 3.9 |
|  | Jaundice | 7 | 3.4 |
|  | Hepatic cyst | 3 | 1.5 |
|  | Hepatic hemangioma | 2 | 1 |
|  | Non-alcoholic steatohepatitis | 1 | 0.5 |
| Gastrointestinal diseases | Chronic gastritis | 19 | 9.3 |
| (n = 44, 21.5%) | Appendicitis | 9 | 4.4 |
|  | Acute pancreatitis | 3 | 1.5 |
|  | Colon polyp | 3 | 1.5 |
|  | Gastrointestinal bleeding | 3 | 1.5 |
|  | Abdominal pain | 2 | 1 |
|  | Gastric polyp | 1 | 0.5 |
|  | Gastric outlet obstruction | 1 | 0.5 |
|  | Rectal polyp | 1 | 0.5 |
|  | Functional constipation | 1 | 0.5 |
|  | Fever | 1 | 0.5 |
| Endocrine/metabolic and other benign conditions | Skin mass | 8 | 3.9 |
| (n = 27, 13.2%) | Thyroid nodule | 5 | 2.4 |
|  | Lipoma | 5 | 2.4 |
|  | Breast nodule | 2 | 1 |
|  | Type 2 diabetes mellitus | 2 | 1 |
|  | Anemia | 1 | 0.5 |
|  | Lymphadenopathy | 1 | 0.5 |
|  | Abnormal uterine bleeding | 1 | 0.5 |
|  | Lower extremity venous insufficiency | 1 | 0.5 |
|  | Melanocytic nevus | 1 | 0.5 |
| Hernia | Inguinal hernia | 11 | 5.4 |
| (n = 11, 5.4%) |  |  |  |
| Renal diseases | Chronic renal insufficiency | 7 | 3.4 |
| (n = 7, 3.4%) |  |  |  |
| Cardiovascular disease | Coronary atherosclerotic heart disease | 1 | 0.5 |
| (n = 1, 0.5%) |  |  |  |
| Other/Unclassified | — | 1 | 0.5 |
| Total |  | 205 | 100 |

# Supplementary Table 2. Routine Blood Indicators

| Routine Blood Indicators | Abbreviation |
| --- | --- |
| Total Protein | TP |
| Albumin | ALB |
| Prealbumin | PAB |
| Globulin | GLB |
| Albumin/Globulin Ratio | A/G |
| Alanine Aminotransferase | ALT |
| Aspartate Aminotransferase | AST |
| AST/ALT Ratio | AST/ALT |
| Gamma-Glutamyl Transferase | GGT |
| Lactate Dehydrogenase | LDH |
| Alkaline Phosphatase | ALP |
| Glutamate Dehydrogenase | GLDH |
| Cholinesterase | CHE |
| Total Bilirubin | TBIL |
| Conjugated Bilirubin | DBIL |
| Total Bile Acids | TBA |
| Glycocholic Acid | GCA |
| Haptoglobin | HP |
| Alpha-1 Antitrypsin | AAT |
| Alpha-1 Acid Glycoprotein | AAG |
| Fibronectin | FN |
| Adenosine Deaminase | ADA |
| Glutathione Reductase | GR |
| Monoamine Oxidase | MAO |
| Creatinine | Cr |
| Urea | Urea |
| Uric Acid | UA |
| Calcium | Ca |
| Corrected Calcium | cCa |
| Inorganic Phosphate | P |
| Magnesium | Mg |
| Cystatin C | Cys-C |
| Retinol-Binding Protein | RBP |
| Complement C1q | C1q |
| Classical Pathway Total Complement Activity | CH50 |
| Potassium | K |
| Sodium | Na |
| Chloride | Cl |
| Total CO₂ / CO₂ Combining Power | TCO2 |
| Anion Gap | AG |
| Triglycerides | TG |
| Total Cholesterol | TC |
| HDL Cholesterol | HDL-C |
| LDL Cholesterol | LDL-C |
| VLDL Cholesterol | VLDL-C |
| Apolipoprotein A1 | ApoA1 |
| Apolipoprotein B | ApoB |
| Apolipoprotein E | ApoE |
| Lipoprotein(a) | Lp(a) |
| Free Fatty Acids | FFA |
| Apolipoprotein A2 | ApoA2 |
| Apolipoprotein C2 | ApoC2 |
| Apolipoprotein C3 | ApoC3 |
| Small Dense LDL Cholesterol | sdLDL-C |
| Creatine Kinase | CK |
| α-Hydroxybutyrate Dehydrogenase | α-HBDH |
| LDH/α-HBDH Ratio | LDH/α-HBDH |
| Homocysteine | Hcy |
| Nitric Oxide | NO |
| Superoxide Dismutase | SOD |
| Glucose | Glu |
| Glycated Albumin | GA |
| Albumin | ALB |
| Alpha-1 Globulin | α1-G |
| Alpha-2 Globulin | α2-G |
| Beta-1 Globulin | β1-G |
| Beta-2 Globulin | β2-G |
| Gamma Globulin | γ-G |
| Helicobacter pylori Antibody | H. pylori Ab |
| eGFR (EPI equation) | eGFR-EPI |
| White Blood Cell Count | WBC |
| Neutrophil Percentage | NEU% |
| Neutrophil Count | NEU# |
| Lymphocyte Percentage | LYM% |
| Lymphocyte Count | LYM# |
| Monocyte Percentage | MONO% |
| Monocyte Count | MONO# |
| Eosinophil Percentage | EOS% |
| Eosinophil Count | EOS# |
| Basophil Percentage | BASO% |
| Basophil Count | BASO# |
| Red Blood Cell Count | RBC |
| Hemoglobin | HGB |
| Hematocrit | HCT |
| Mean Corpuscular Volume | MCV |
| Mean Corpuscular Hemoglobin | MCH |
| Mean Corpuscular Hemoglobin Concentration | MCHC |
| Red Cell Distribution Width | RDW |
| Platelet Count | PLT |
| Mean Platelet Volume | MPV |
| High-Sensitivity C-Reactive Protein | hs-CRP |
| D-dimer | D-dimer |

# Supplementary Table 3 Details of 26 quantitative features extracted from tongue images.

| **English Term** | **Abbreviation** | **Explanation** |
| --- | --- | --- |
| Tongue Coating Color – L* (Lightness) | TC-L | Lightness in the CIE-Lab color space; lower values indicate darker tones, while higher values indicate lighter tones. |
| Tongue Coating Color – a* (Green–Red Axis) | TC-A | The a* axis in the CIE-Lab color space; higher values indicate redder tones, lower values indicate greener tones. |
| Tongue Coating Color – b* (Blue–Yellow Axis) | TC-B | The b* axis in the CIE-Lab color space; higher values indicate yellowish tones, while lower values reflect bluish tones. |
| Degree of Black Coating | TC-Black | Degree of black coating on the tongue surface, expressed on a scale from 0 to 1. |
| Degree of White Coating | TC-White | Degree of white coating on the tongue surface, expressed on a scale from 0 to 1. |
| Degree of Yellow Coating | TC-Yellow | Degree of yellow coating on the tongue surface, expressed on a scale from 0 to 1. |
| Tongue Body Color – L* | TB-L | Lightness of the tongue body in the CIE-Lab color space; lower values indicate darker tones, higher values indicate lighter tones. |
| Tongue Body Color – a* | TB-A | The a* axis of the tongue body; higher values indicate redness, lower values reflect greenness. |
| Tongue Body Color – b* | TB-B | The b* axis of the tongue body; higher values indicate yellowish tones, lower values indicate bluish tones. |
| Full Tongue Color – L* | FT-L | Lightness of the entire tongue in the CIE-Lab space; lower values indicate darker tones, higher values indicate lighter tones. |
| Full Tongue Color – a* | FT-A | The a* axis for the whole tongue; high values indicate red tones, low values indicate green tones. |
| Full Tongue Color – b* | FT-B | The b* axis for the whole tongue; high values indicate yellow, low values indicate blue. |
| Qualitative Tongue Color Classification | TCC | Qualitative tongue-body color classification: 3 = pale-white, 0 = light red, 1 = red, 2 = deep red (crimson), 4 = purple. TCC4 denotes the category coded as 4 (purple). |
| Coating Color Classification | CCC | Coating color classification: 0 = white, 1 = mixed white–yellow, 2 = yellow, 3 = gray–black. |
| Tongue Prickles | Prick | Prickles are defined as red papillary spots on the tongue surface; fewer than 20 = absent (0), ≥20 = present (1). |
| Quantitative Prickle Count | Prick-Quant | Quantitative count of red prickles on the tongue surface. |
| Tongue Fissures | Crack | Fissures covering ≤0.4% of the tongue area are classified as absent (0), while >0.4% are classified as present (1). |
| Quantitative Fissure Area | Crack-Quant | Percentage of tongue surface area affected by fissures. |
| Teeth Marks | TM | Teeth marks ≤2 are classified as absent (0); >2 are classified as present (1). |
| Quantitative Teeth-Mark Count | TM-Quant | Number of teeth marks on the tongue margin. |
| Tongue Body Shape | TBS | Ratio of tongue width to length: <0.7 = thin tongue, 0.7–0.99 = normal, ≥1.0 = enlarged (puffy) tongue; coded as 1 = thin, 0 = normal, 2 = enlarged. |
| Quantitative Body-Shape Index | TBS-Quant | Width-to-length ratio of the tongue body. |
| Coating Thickness | CT | Coating thickness index: 0 = none, >0–0.58 = scant, 0.58–0.78 = thin, >0.78 = thick; coded as 1 = none, 2 = scant, 0 = thin, 3 = thick. |
| Quantitative Coating Thickness Index | CT-Quant | Quantitative tongue coating thickness index. |
| Greasiness of Tongue Coating | Greasy | Greasiness index: 0–0.5 = non-greasy (0), >0.5–0.8 = greasy (1), ≥0.8 = heavily greasy (2). |
| Quantitative Greasiness Index | Greasy-Quant | Quantitative greasiness index of the tongue coating. |

# Supplementary Table 4. Details of eight diagnostic pulse elements extracted from pulse features.

| **English Term** | **Abbreviation** | **Explanation** |
| --- | --- | --- |
| Left Pulse Strength | LPS | An index reflecting the strength of pulse beat force. |
| Left Vein Power | LVPow | An indicator of the pulse beat force, categorized as weak, normal, or strong. |
| Left Vein Position | LVPos | Depth of pulse presentation, categorized as superficial, moderate, or deep. |
| Left Vein Rate | LVR | Number of pulse beats per minute. |
| Left Vein Fluency | LVF | Smoothness perceived during palpation, categorized as slippery, normal, or choppy. |
| Left Vein Tension | LVT | Degree of vascular tension, categorized as normal, wiry, or tight. |
| Left Vein Rhythm | LVRhy | Indicator of pulse rhythm regularity, categorized as regular or irregular. |
| Left Healthy Value | LHV | Composite score based on left-hand pulse indices. |
| Right Pulse Strength | RPS | An index reflecting the strength of pulse beat force. |
| Right Vein Power | RVPow | An indicator of the pulse beat force, categorized as weak, normal, or strong. |
| Right Vein Position | RVPos | Depth of pulse presentation, categorized as superficial, moderate, or deep. |
| Right Vein Rate | RVR | Number of pulse beats per minute. |
| Right Vein Fluency | RVF | Smoothness perceived during palpation, categorized as slippery, normal, or choppy. |
| Right Vein Tension | RVT | Degree of vascular tension, categorized as normal, wiry, or tight. |
| Right Vein Rhythm | RVRhy | Indicator of pulse rhythm regularity, categorized as regular or irregular. |
| Right Healthy Value | RHV | Composite score based on right-hand pulse indices. |

# Supplementary Table 5. Explanations of nine TCM constitution types.

| **Constitution Score** | **Abbreviation** | **Constitution Feature** |
| --- | --- | --- |
| **Balanced Constitution Score** | **BC-Score** | A constitution characterized by harmonized Yin–Yang and Qi–Blood, typically presenting with moderate body shape, rosy complexion, and abundant vitality. |
| **Qi-Deficiency Constitution Score** | **QDC-Score** | A constitution marked by insufficiency of vital Qi, often manifested as fatigue, shortness of breath, spontaneous sweating, and general weakness. |
| **Yang-Deficiency Constitution Score** | **YDC-Score** | A constitution characterized by a deficiency of Yang Qi, with symptoms such as intolerance to cold, cold extremities, and signs of internal coldness. |
| **Yin-Deficiency Constitution Score** | **IDC-Score** | A constitution associated with depletion of Yin fluids, typically presenting with dry mouth and throat, warm palms and soles, and symptoms of internal heat. |
| **Phlegm-Dampness Constitution Score** | **PDC-Score** | A constitution involving internal accumulation of phlegm and dampness, often presenting with obesity, abdominal fullness, sticky mouth, and greasy tongue coating. |
| **Damp-Heat Constitution Score** | **DHC-Score** | A constitution characterized by internal retention of damp-heat, commonly showing oily facial skin, bitter taste, and yellow-greasy tongue coating. |
| **Blood-Stasis Constitution Score** | **BSC-Score** | A constitution exhibiting impaired blood circulation, often manifested by a dull or dark complexion and a purplish tongue. |
| **Qi-Stagnation Constitution Score** | **QSC-Score** | A constitution with stagnation of Qi movement, usually presenting with emotional depression, irritability, and susceptibility to anxiety. |
| **Inherited/Specific Constitution Score** | **ISC-Score** | A constitution resulting from congenital abnormalities, typically associated with physiological defects, allergic tendencies, or hypersensitivity. |

# Supplementary Table 6.Baseline characteristics of the study participants.

| Variables | Total (n = 514) | lung_cancer (n = 309) | non_cancer (n = 205) | p |
| --- | --- | --- | --- | --- |
| age, Median (Q1,Q3) | 66 (57, 71) | 67 (61, 71) | 60 (47, 69) | < 0.001 |
| sex, n (%) |  |  |  | 0.918 |
| female | 281 (55) | 170 (55) | 111 (54) |  |
| male | 233 (45) | 139 (45) | 94 (46) |  |
| TC.L, Median (Q1,Q3) | 103.17 (96.4, 111.23) | 102.53 (95.57, 109.5) | 105.42 (96.92, 116.46) | 0.001 |
| TC.A, Median (Q1,Q3) | 139.22 (137.68, 141.12) | 139.64 (137.88, 141.34) | 138.68 (137.14, 140.39) | < 0.001 |
| TC.B, Mean ± SD | 105.71 ± 4.59 | 104.78 ± 3.99 | 107.1 ± 5.06 | < 0.001 |
| TC.Black, Median (Q1,Q3) | 0 (0, 0.01) | 0 (0, 0.01) | 0 (0, 0.02) | 0.076 |
| TC.White, Median (Q1,Q3) | 0.13 (0, 0.62) | 0.06 (0, 0.38) | 0.47 (0.02, 0.87) | < 0.001 |
| TC.Yellow, Median (Q1,Q3) | 0.4 (0.03, 0.89) | 0.6 (0.13, 0.91) | 0.12 (0, 0.77) | < 0.001 |
| TB.L, Mean ± SD | 124.85 ± 13.98 | 122.22 ± 12.86 | 128.81 ± 14.68 | < 0.001 |
| TB.A, Median (Q1,Q3) | 135.74 (134.13, 137.79) | 135.81 (134.29, 137.97) | 135.64 (133.84, 137.68) | 0.179 |
| TB.B, Mean ± SD | 111.2 ± 4.41 | 110.23 ± 4.02 | 112.65 ± 4.58 | < 0.001 |
| FT.L, Mean ± SD | 49.5 ± 4.98 | 48.83 ± 4.58 | 50.52 ± 5.38 | < 0.001 |
| FT.A, Median (Q1,Q3) | 14.68 (12.79, 17.46) | 15.53 (13.41, 18.09) | 13.56 (11.73, 16.2) | < 0.001 |
| FT.B, Median (Q1,Q3) | 4.32 (2.52, 5.96) | 4.84 (3.54, 6.27) | 3.21 (1.11, 5.3) | < 0.001 |
| TCC, n (%) |  |  |  | < 0.001 |
| 0 | 335 (65) | 231 (75) | 104 (51) |  |
| 1 | 63 (12) | 51 (17) | 12 (6) |  |
| 2 | 3 (1) | 0 (0) | 3 (1) |  |
| 3 | 59 (11) | 19 (6) | 40 (20) |  |
| 4 | 54 (11) | 8 (3) | 46 (22) |  |
| CCC, n (%) |  |  |  | 0.019 |
| 0 | 124 (24) | 71 (23) | 53 (26) |  |
| 1 | 311 (61) | 199 (64) | 112 (55) |  |
| 2 | 38 (7) | 23 (7) | 15 (7) |  |
| 3 | 41 (8) | 16 (5) | 25 (12) |  |
| Prick, n (%) |  |  |  | 0.028 |
| 0 | 260 (51) | 169 (55) | 91 (44) |  |
| 1 | 254 (49) | 140 (45) | 114 (56) |  |
| Prick.Quant, Median (Q1,Q3) | 10 (3, 22) | 8 (2, 20) | 13 (5, 25) | 0.002 |
| Crack, n (%) |  |  |  | 0.002 |
| 0 | 292 (57) | 158 (51) | 134 (65) |  |
| 1 | 222 (43) | 151 (49) | 71 (35) |  |
| Crack.Quant, Median (Q1,Q3) | 0.3 (0, 0.83) | 0.38 (0.02, 0.87) | 0.17 (0, 0.67) | < 0.001 |
| TM, n (%) |  |  |  | 0.019 |
| 0 | 257 (50) | 168 (54) | 89 (43) |  |
| 1 | 257 (50) | 141 (46) | 116 (57) |  |
| TM.Quant, Median (Q1,Q3) | 1.5 (0, 3) | 1 (0, 3) | 2 (1, 3) | 0.003 |
| TBS, n (%) |  |  |  | < 0.001 |
| 0 | 254 (49) | 176 (57) | 78 (38) |  |
| 1 | 100 (19) | 51 (17) | 49 (24) |  |
| 2 | 160 (31) | 82 (27) | 78 (38) |  |
| TBS.Quant, Median (Q1,Q3) | 0.85 (0.83, 1.2) | 0.85 (0.83, 1.19) | 0.85 (0.81, 1.21) | 0.951 |
| CT, n (%) |  |  |  | < 0.001 |
| 0 | 163 (32) | 123 (40) | 40 (20) |  |
| 2 | 2 (0) | 1 (0) | 1 (0) |  |
| 3 | 349 (68) | 185 (60) | 164 (80) |  |
| CT.Quant, Median (Q1,Q3) | 0.81 (0.69, 0.89) | 0.78 (0.67, 0.86) | 0.86 (0.75, 0.92) | < 0.001 |
| Greasy, n (%) |  |  |  | 0.12 |
| 0 | 28 (5) | 13 (4) | 15 (7) |  |
| 1 | 485 (94) | 296 (96) | 189 (92) |  |
| 2 | 1 (0) | 0 (0) | 1 (0) |  |
| Greasy.Quant, Median (Q1,Q3) | 0.72 (0.67, 0.75) | 0.72 (0.67, 0.75) | 0.72 (0.67, 0.75) | 0.222 |
| LPS, Median (Q1,Q3) | 11.48 (7.07, 16.78) | 11 (7.06, 16.74) | 12.33 (7.36, 16.8) | 0.285 |
| LVPow, n (%) |  |  |  | 0.858 |
| 0 | 1 (0) | 1 (0) | 0 (0) |  |
| 1 | 124 (24) | 75 (24) | 49 (24) |  |
| 2 | 282 (55) | 172 (56) | 110 (54) |  |
| 3 | 107 (21) | 61 (20) | 46 (22) |  |
| LVPos, n (%) |  |  |  | 0.141 |
| 1 | 182 (35) | 99 (32) | 83 (40) |  |
| 2 | 317 (62) | 201 (65) | 116 (57) |  |
| 3 | 15 (3) | 9 (3) | 6 (3) |  |
| LVR, Median (Q1,Q3) | 79 (70, 89) | 80 (71, 89) | 77 (68, 88) | 0.086 |
| LVF, n (%) |  |  |  | 0.209 |
| 1 | 6 (1) | 4 (1) | 2 (1) |  |
| 2 | 479 (93) | 292 (94) | 187 (91) |  |
| 3 | 29 (6) | 13 (4) | 16 (8) |  |
| LVT, n (%) |  |  |  | 0.474 |
| 1 | 219 (43) | 126 (41) | 93 (45) |  |
| 2 | 212 (41) | 134 (43) | 78 (38) |  |
| 3 | 83 (16) | 49 (16) | 34 (17) |  |
| LVRhy, n (%) |  |  |  | 1 |
| 1 | 500 (97) | 301 (97) | 199 (97) |  |
| 2 | 14 (3) | 8 (3) | 6 (3) |  |
| LHV, n (%) |  |  |  | 0.22 |
| 60 | 3 (1) | 3 (1) | 0 (0) |  |
| 65 | 2 (0) | 0 (0) | 2 (1) |  |
| 70 | 13 (3) | 10 (3) | 3 (1) |  |
| 75 | 24 (5) | 10 (3) | 14 (7) |  |
| 80 | 126 (25) | 80 (26) | 46 (22) |  |
| 85 | 88 (17) | 55 (18) | 33 (16) |  |
| 90 | 218 (42) | 129 (42) | 89 (43) |  |
| 95 | 31 (6) | 17 (6) | 14 (7) |  |
| 100 | 9 (2) | 5 (2) | 4 (2) |  |
| RPS, Median (Q1,Q3) | 11.42 (6.89, 16.19) | 11.01 (7.23, 15.7) | 11.79 (5.88, 16.45) | 0.962 |
| RVPow, n (%) |  |  |  | 0.458 |
| 1 | 132 (26) | 75 (24) | 57 (28) |  |
| 2 | 288 (56) | 180 (58) | 108 (53) |  |
| 3 | 94 (18) | 54 (17) | 40 (20) |  |
| RVPos, n (%) |  |  |  | 0.287 |
| 1 | 164 (32) | 92 (30) | 72 (35) |  |
| 2 | 336 (65) | 210 (68) | 126 (61) |  |
| 3 | 14 (3) | 7 (2) | 7 (3) |  |
| RVR, Median (Q1,Q3) | 79 (70, 88) | 80 (71, 89) | 77 (69, 87) | 0.028 |
| RVF, n (%) |  |  |  | 0.392 |
| 1 | 9 (2) | 5 (2) | 4 (2) |  |
| 2 | 467 (91) | 285 (92) | 182 (89) |  |
| 3 | 38 (7) | 19 (6) | 19 (9) |  |
| RVT, n (%) |  |  |  | 0.004 |
| 1 | 237 (46) | 124 (40) | 113 (55) |  |
| 2 | 217 (42) | 145 (47) | 72 (35) |  |
| 3 | 60 (12) | 40 (13) | 20 (10) |  |
| RVRhy, n (%) |  |  |  | 0.771 |
| 1 | 502 (98) | 301 (97) | 201 (98) |  |
| 2 | 12 (2) | 8 (3) | 4 (2) |  |
| RHV, n (%) |  |  |  | 0.08 |
| 60 | 1 (0) | 1 (0) | 0 (0) |  |
| 65 | 4 (1) | 2 (1) | 2 (1) |  |
| 70 | 13 (3) | 11 (4) | 2 (1) |  |
| 75 | 21 (4) | 12 (4) | 9 (4) |  |
| 80 | 124 (24) | 84 (27) | 40 (20) |  |
| 85 | 77 (15) | 41 (13) | 36 (18) |  |
| 90 | 233 (45) | 140 (45) | 93 (45) |  |
| 95 | 34 (7) | 15 (5) | 19 (9) |  |
| 100 | 7 (1) | 3 (1) | 4 (2) |  |
| BC.Score, Median (Q1,Q3) | 68.75 (50, 81.25) | 62.5 (50, 81.25) | 75 (50, 87.5) | 0.023 |
| QDC.Score, Median (Q1,Q3) | 25 (12.5, 43.75) | 31.25 (18.75, 50) | 18.75 (8, 31.25) | < 0.001 |
| YDC.Score, Median (Q1,Q3) | 16.67 (0, 41.67) | 16.67 (0, 41.67) | 16.67 (0, 33.33) | 0.045 |
| IDC.Score, Median (Q1,Q3) | 25 (12.5, 37.5) | 25 (12.5, 43.75) | 18.75 (2, 31.25) | < 0.001 |
| PDC.Score, Median (Q1,Q3) | 25 (12.5, 37.5) | 31.25 (12.5, 43.75) | 25 (12.5, 37.5) | 0.023 |
| DHC.Score, Median (Q1,Q3) | 25 (8.33, 41.67) | 25 (16.67, 41.67) | 16.67 (8.33, 33.33) | < 0.001 |
| BSC.Score, Median (Q1,Q3) | 25 (12.5, 37.5) | 31.25 (12.5, 43.75) | 18.75 (0, 31.25) | < 0.001 |
| QSC.Score, Median (Q1,Q3) | 12.5 (0, 31.25) | 12.5 (0, 31.25) | 6.25 (0, 25) | 0.039 |
| TP, Median (Q1,Q3) | 66.1 (62.4, 69.2) | 65.4 (62.3, 69.3) | 66.59 (62.6, 68.9) | 0.283 |
| ALB, Median (Q1,Q3) | 38.09 (36.5, 40.1) | 38.2 (36.6, 40) | 38 (36.4, 40.4) | 0.978 |
| PAB, Median (Q1,Q3) | 0.2 (0.18, 0.23) | 0.21 (0.18, 0.23) | 0.2 (0.18, 0.22) | 0.013 |
| GLB, Median (Q1,Q3) | 27.17 (25.2, 30) | 27.1 (24.7, 30) | 27.17 (25.8, 30) | 0.53 |
| A.G, Median (Q1,Q3) | 1.38 (1.27, 1.52) | 1.4 (1.26, 1.54) | 1.37 (1.28, 1.47) | 0.043 |
| ALT, Median (Q1,Q3) | 20 (14.25, 28) | 18 (14, 26) | 23.73 (15, 31.52) | < 0.001 |
| AST, Median (Q1,Q3) | 21 (17, 25) | 20 (17, 24) | 22 (18, 27) | 0.001 |
| AST.ALT, Median (Q1,Q3) | 1 (0.87, 1.3) | 1.07 (0.88, 1.31) | 0.96 (0.85, 1.26) | 0.008 |
| GGT, Median (Q1,Q3) | 25 (17, 36.92) | 22 (17, 32) | 30.7 (19, 46.05) | < 0.001 |
| LDH, Median (Q1,Q3) | 177.2 (157, 197) | 175 (157, 202) | 178.26 (158, 191) | 0.764 |
| ALP, Median (Q1,Q3) | 70.36 (59, 84.78) | 69.28 (57, 84) | 71.78 (65, 87) | 0.018 |
| GLDH, Median (Q1,Q3) | 3 (1.7, 4.82) | 2.7 (1.8, 4.4) | 3.5 (1.7, 5.35) | 0.003 |
| CHE, Median (Q1,Q3) | 8203.14 (7270, 9154.5) | 8281 (7303, 9382) | 8129.89 (7142, 8927) | 0.051 |
| TBIL, Median (Q1,Q3) | 15.38 (12.7, 18.3) | 14.7 (12.5, 17.3) | 15.89 (13.3, 20.6) | 0.002 |
| DBIL, Median (Q1,Q3) | 2.43 (1.9, 3.2) | 2.3 (1.8, 3) | 2.7 (2.3, 3.9) | < 0.001 |
| TBA, Median (Q1,Q3) | 3.8 (2.4, 5.7) | 3.5 (2.2, 5.55) | 4.52 (2.5, 6.07) | 0.041 |
| GCA, Median (Q1,Q3) | 1.49 (1.2, 1.9) | 1.36 (1.13, 1.8) | 1.57 (1.34, 2.05) | < 0.001 |
| HP, Median (Q1,Q3) | 0.88 (0.6, 1.18) | 0.88 (0.54, 1.22) | 0.87 (0.7, 1.12) | 0.676 |
| AAT, Median (Q1,Q3) | 1.16 (1.06, 1.3) | 1.16 (1.05, 1.27) | 1.15 (1.1, 1.35) | 0.055 |
| AAG, Median (Q1,Q3) | 0.63 (0.55, 0.77) | 0.63 (0.54, 0.76) | 0.63 (0.56, 0.78) | 0.506 |
| FN, Median (Q1,Q3) | 325.04 (281, 373) | 331 (287, 380) | 322 (268, 361) | 0.006 |
| ADA, Median (Q1,Q3) | 10.89 (9, 13) | 10 (9, 13) | 11 (9.06, 14) | 0.004 |
| GR, Median (Q1,Q3) | 62.12 (56.25, 70) | 63 (56, 72) | 62 (57, 68) | 0.352 |
| MAO, Median (Q1,Q3) | 6.72 (5.8, 7.6) | 6.6 (5.6, 7.9) | 6.77 (6.32, 7.28) | 0.319 |
| Cr, Median (Q1,Q3) | 64.2 (55.15, 73.47) | 63.3 (53.5, 73.3) | 65.4 (58.1, 75) | 0.02 |
| Urea, Median (Q1,Q3) | 5.15 (4.42, 5.9) | 5.1 (4.43, 5.97) | 5.22 (4.37, 5.81) | 0.98 |
| UA, Median (Q1,Q3) | 304 (255.25, 351.52) | 304 (259, 356) | 306.29 (246, 342) | 0.073 |
| Ca, Median (Q1,Q3) | 2.3 (2.23, 2.36) | 2.3 (2.24, 2.36) | 2.29 (2.21, 2.34) | 0.035 |
| cCa, Median (Q1,Q3) | 2.33 (2.28, 2.37) | 2.34 (2.29, 2.38) | 2.33 (2.27, 2.35) | < 0.001 |
| P, Median (Q1,Q3) | 1.13 (1.02, 1.23) | 1.14 (1.03, 1.24) | 1.12 (1.01, 1.21) | 0.206 |
| Mg, Median (Q1,Q3) | 0.86 (0.82, 0.89) | 0.86 (0.82, 0.9) | 0.85 (0.82, 0.89) | 0.205 |
| Cys.C, Median (Q1,Q3) | 1.02 (0.92, 1.15) | 1.02 (0.92, 1.16) | 1.03 (0.93, 1.13) | 0.771 |
| RBP, Median (Q1,Q3) | 43.4 (36.32, 50.3) | 43.8 (37.2, 52.4) | 43.1 (33.8, 47.3) | 0.004 |
| C1q, Median (Q1,Q3) | 170.42 (149, 199) | 169 (145, 200) | 173.67 (159, 194) | 0.057 |
| CH50, Median (Q1,Q3) | 50 (45, 54) | 50 (44, 55) | 50.47 (46, 52.69) | 0.599 |
| K, Median (Q1,Q3) | 3.95 (3.74, 4.1) | 3.95 (3.78, 4.16) | 3.95 (3.65, 4.02) | < 0.001 |
| Na, Median (Q1,Q3) | 140.9 (139.8, 141.9) | 140.8 (139.7, 142) | 140.92 (140, 141.6) | 0.858 |
| Cl, Median (Q1,Q3) | 106.16 (104.7, 107.38) | 106.2 (104.7, 107.7) | 106.1 (104.71, 106.7) | 0.11 |
| TCO2, Median (Q1,Q3) | 24.95 (23.7, 26.8) | 25.4 (23.8, 27.2) | 24.5 (23.66, 26.1) | < 0.001 |
| AG, Median (Q1,Q3) | 9.53 (8.22, 10.6) | 9.1 (7.8, 10.34) | 9.9 (9.26, 11.2) | < 0.001 |
| TG, Median (Q1,Q3) | 1.28 (0.96, 1.58) | 1.3 (0.98, 1.74) | 1.27 (0.94, 1.42) | 0.015 |
| TC, Median (Q1,Q3) | 4.53 (3.99, 5.16) | 4.65 (3.96, 5.32) | 4.5 (4.15, 4.78) | 0.026 |
| HDL.C, Median (Q1,Q3) | 1.28 (1.09, 1.45) | 1.28 (1.08, 1.48) | 1.27 (1.17, 1.35) | 0.624 |
| LDL.C, Median (Q1,Q3) | 2.94 (2.53, 3.39) | 2.96 (2.47, 3.49) | 2.93 (2.67, 3.16) | 0.242 |
| VLDL.C, Median (Q1,Q3) | 0.34 (0.2, 0.45) | 0.37 (0.22, 0.5) | 0.32 (0.14, 0.38) | < 0.001 |
| ApoA1, Median (Q1,Q3) | 1.16 (1.02, 1.26) | 1.14 (1.01, 1.27) | 1.18 (1.04, 1.23) | 0.869 |
| ApoB, Mean ± SD | 1.03 ± 0.25 | 1.03 ± 0.26 | 1.03 ± 0.23 | 0.923 |
| ApoE, Median (Q1,Q3) | 34.98 (29.42, 39.87) | 35.5 (29.3, 41.3) | 34.1 (29.6, 38.2) | 0.109 |
| Lp.a., Median (Q1,Q3) | 174.12 (127.1, 262.82) | 175 (134, 285) | 171 (111, 234.96) | 0.05 |
| FFA, Median (Q1,Q3) | 0.38 (0.29, 0.49) | 0.36 (0.28, 0.48) | 0.39 (0.34, 0.51) | 0.005 |
| ApoA2, Median (Q1,Q3) | 0.31 (0.27, 0.34) | 0.3 (0.27, 0.34) | 0.32 (0.28, 0.34) | 0.114 |
| ApoC2, Median (Q1,Q3) | 0.04 (0.03, 0.04) | 0.04 (0.03, 0.04) | 0.04 (0.03, 0.04) | 0.002 |
| ApoC3, Median (Q1,Q3) | 0.08 (0.07, 0.1) | 0.09 (0.07, 0.1) | 0.08 (0.06, 0.09) | < 0.001 |
| sdLDL.C, Median (Q1,Q3) | 0.29 (0.22, 0.36) | 0.29 (0.22, 0.39) | 0.29 (0.23, 0.32) | 0.083 |
| CK, Median (Q1,Q3) | 78 (61.2, 105.72) | 77.4 (57.9, 106.6) | 79.5 (67.1, 102.53) | 0.543 |
| α.HBDH, Median (Q1,Q3) | 107.34 (97, 120.44) | 108 (95, 124) | 107.05 (100.94, 116) | 0.835 |
| LDH.α.HBDH, Median (Q1,Q3) | 1.61 (1.6, 1.7) | 1.6 (1.6, 1.7) | 1.63 (1.6, 1.7) | 0.817 |
| Hcy, Median (Q1,Q3) | 9 (7.7, 10.52) | 8.9 (7.5, 10.44) | 9.09 (8.07, 10.7) | 0.08 |
| NO, Median (Q1,Q3) | 31.9 (29.13, 35.98) | 32.5 (29, 36.2) | 31.6 (29.7, 34.7) | 0.213 |
| SOD, Median (Q1,Q3) | 168.11 (158.02, 178.25) | 166.6 (157.01, 177.7) | 169.6 (158.6, 179) | 0.087 |
| Glu, Median (Q1,Q3) | 5.06 (4.69, 5.61) | 5.08 (4.67, 5.68) | 5.05 (4.73, 5.45) | 0.588 |
| GA, Median (Q1,Q3) | 15.56 (14.52, 17.15) | 15.63 (14.54, 17.19) | 15.43 (14.52, 17.08) | 0.238 |
| ALB.1, Median (Q1,Q3) | 38.2 (36.5, 40.1) | 38.2 (36.6, 40) | 38.2 (36.4, 40.4) | 0.8 |
| α1.G, Median (Q1,Q3) | 3.6 (3.3, 4.1) | 3.6 (3.3, 4) | 3.59 (3.4, 4.3) | 0.245 |
| α2.G, Median (Q1,Q3) | 8.48 (7.8, 9.5) | 8.6 (7.6, 9.5) | 8.4 (8, 9.6) | 0.906 |
| β1.G, Median (Q1,Q3) | 5.53 (5.2, 5.8) | 5.5 (5.2, 5.8) | 5.56 (5.4, 5.78) | 0.175 |
| β2.G, Median (Q1,Q3) | 5.3 (4.8, 5.8) | 5.3 (4.8, 5.8) | 5.28 (4.9, 5.8) | 0.64 |
| γ.G, Median (Q1,Q3) | 16.6 (14.7, 18.2) | 16.4 (14.3, 18.3) | 16.71 (15.7, 18) | 0.024 |
| H..pylori.Ab, Median (Q1,Q3) | 9.25 (3.62, 20.74) | 6.3 (2.9, 15.3) | 14.8 (8, 23.3) | < 0.001 |
| eGFR.EPI, Median (Q1,Q3) | 93.18 (85.32, 100.36) | 92.86 (84.66, 100.27) | 93.85 (86.3, 101.15) | 0.134 |
| WBC, Median (Q1,Q3) | 5.54 (4.59, 6.58) | 5.12 (4.26, 6.4) | 5.74 (5.08, 6.97) | < 0.001 |
| NEU., Median (Q1,Q3) | 58.21 (53, 65.3) | 58.9 (52.5, 66) | 58.02 (53.6, 64.1) | 0.896 |
| NEU..1, Median (Q1,Q3) | 3.15 (2.53, 4.05) | 2.96 (2.46, 3.86) | 3.25 (2.89, 4.28) | < 0.001 |
| LYM., Median (Q1,Q3) | 30.3 (23.33, 35.38) | 29.4 (22.9, 35.8) | 30.78 (25, 34.1) | 0.557 |
| LYM..1, Median (Q1,Q3) | 1.6 (1.2, 1.94) | 1.5 (1.17, 1.8) | 1.84 (1.4, 2.07) | < 0.001 |
| MONO., Median (Q1,Q3) | 7.9 (6.7, 9.3) | 8 (6.8, 9.7) | 7.8 (6.6, 8.5) | 0.033 |
| MONO..1, Median (Q1,Q3) | 0.44 (0.35, 0.51) | 0.42 (0.33, 0.49) | 0.45 (0.4, 0.52) | < 0.001 |
| EOS., Median (Q1,Q3) | 2.32 (1.5, 3.27) | 2.4 (1.6, 3.5) | 2.3 (1.4, 2.9) | 0.02 |
| EOS..1, Median (Q1,Q3) | 0.12 (0.08, 0.17) | 0.12 (0.08, 0.19) | 0.12 (0.08, 0.16) | 0.347 |
| BASO., Median (Q1,Q3) | 0.5 (0.34, 0.7) | 0.5 (0.4, 0.7) | 0.5 (0.3, 0.6) | 0.021 |
| BASO..1, Median (Q1,Q3) | 0.03 (0.02, 0.04) | 0.03 (0.02, 0.04) | 0.03 (0.02, 0.03) | 0.785 |
| RBC, Median (Q1,Q3) | 4.2 (3.91, 4.52) | 4.18 (3.86, 4.48) | 4.21 (4, 4.58) | 0.103 |
| HGB, Median (Q1,Q3) | 127 (119, 137) | 126 (118, 136) | 127.87 (122, 138) | 0.085 |
| HCT, Median (Q1,Q3) | 38.3 (35.8, 40.9) | 38 (35.5, 40.8) | 38.4 (36.8, 41.1) | 0.164 |
| MCV, Median (Q1,Q3) | 91 (89, 94) | 91.47 (89, 94) | 90.79 (89, 93) | 0.07 |
| MCH, Median (Q1,Q3) | 30.4 (29.5, 31.37) | 30.44 (29.4, 31.4) | 30.39 (29.6, 31.3) | 0.924 |
| MCHC, Median (Q1,Q3) | 333 (327, 339) | 331.75 (325, 339) | 333.78 (328, 338) | 0.012 |
| RDW, Median (Q1,Q3) | 12.8 (12.4, 13.4) | 12.81 (12.3, 13.44) | 12.8 (12.55, 13.4) | 0.677 |
| PLT, Median (Q1,Q3) | 199.35 (164.25, 233.75) | 194 (161, 231) | 204 (175, 237) | 0.063 |
| MPV, Median (Q1,Q3) | 10.33 (9.8, 10.91) | 10.3 (9.8, 11) | 10.37 (9.8, 10.9) | 0.739 |
| hs.CRP, Median (Q1,Q3) | 0.84 (0.5, 1.84) | 0.8 (0.5, 1.69) | 0.93 (0.63, 2.48) | 0.002 |
| D.dimer, Median (Q1,Q3) | 0.34 (0.21, 0.61) | 0.32 (0.22, 0.61) | 0.36 (0.19, 0.61) | 0.757 |

# Supplementary Table 7. Baseline characteristics of lung cancer patients (N = 309)

| Variable | Value |
| --- | --- |
| Total cases | 309 |
| Sex |  |
| Male | 139 (45.0%) |
| Female | 170 (55.0%) |
| Age |  |
| Mean | 66.1 years |
| Surgery status | |
| Surgery performed | 166 (53.7%) |
| No surgery | 143 (46.3%) |
| Tumor stage | |
| Stage I | 173 (56.0%) |
| Stage II | 32 (10.4%) |
| Stage III | 53 (17.2%) |
| Stage IV | 51 (16.5%) |
| Pathologic type | |
| Adenocarcinoma | 272 (88.0%) |
| Squamous cell carcinoma | 21 (6.8%) |
| Other | 16 (5.2%) |

# Supplementary Table 8. Net benefit of six predictive models across clinically relevant threshold probabilities.

| threshold | Model | Net_Benefit | CI |
| --- | --- | --- | --- |
| 0.1 | LR | 0.4684 | 0.386–0.5513 |
| 0.15 | LR | 0.4589 | 0.3754–0.5451 |
| 0.2 | LR | 0.449 | 0.362–0.5373 |
| 0.25 | LR | 0.4189 | 0.3333–0.5022 |
| 0.3 | LR | 0.3725 | 0.282–0.4666 |
| 0.1 | GBM | 0.4583 | 0.3752–0.5426 |
| 0.15 | GBM | 0.4393 | 0.3502–0.5233 |
| 0.2 | GBM | 0.4261 | 0.3344–0.5114 |
| 0.25 | GBM | 0.4142 | 0.3203–0.5043 |
| 0.3 | GBM | 0.3864 | 0.2941–0.4787 |
| 0.1 | XGBoost | 0.4651 | 0.3802–0.5491 |
| 0.15 | XGBoost | 0.4448 | 0.3568–0.5321 |
| 0.2 | XGBoost | 0.4296 | 0.3409–0.5195 |
| 0.25 | XGBoost | 0.4186 | 0.3268–0.513 |
| 0.3 | XGBoost | 0.4033 | 0.307–0.4973 |
| 0.1 | MLP | 0.4217 | 0.342–0.5022 |
| 0.15 | MLP | 0.4059 | 0.327–0.4867 |
| 0.2 | MLP | 0.3941 | 0.3117–0.4773 |
| 0.25 | MLP | 0.3761 | 0.2922–0.4632 |
| 0.3 | MLP | 0.3576 | 0.2718–0.4453 |
| 0.1 | RF | 0.4512 | 0.3644–0.5404 |
| 0.15 | RF | 0.4333 | 0.3419–0.5237 |
| 0.2 | RF | 0.4231 | 0.3295–0.5147 |
| 0.25 | RF | 0.406 | 0.3117–0.5 |
| 0.3 | RF | 0.3879 | 0.2903–0.4833 |
| 0.1 | SVM | 0.4547 | 0.3694–0.5411 |
| 0.15 | SVM | 0.4356 | 0.3491–0.5294 |
| 0.2 | SVM | 0.4114 | 0.3246–0.4984 |
| 0.25 | SVM | 0.3861 | 0.2986–0.4741 |
| 0.3 | SVM | 0.3628 | 0.269–0.4555 |

Net benefit values were derived from decision curve analysis on the independent validation set (n = 156). Bootstrap 95% confidence intervals (CIs) were estimated from 1,000 resampling iterations. LR, logistic regression; GBM, gradient boosting machine; MLP, multilayer perceptron; RF, random forest; SVM, support vector machine.
